# Updates in combined approaches of radiotherapy and immune checkpoint inhibitors for the treatment of breast cancer

**DOI:** 10.3389/fonc.2022.1022542

**Published:** 2022-10-26

**Authors:** Kassidy M. Jungles, Erin A. Holcomb, Ashley N. Pearson, Kalli R. Jungles, Caroline R. Bishop, Lori J. Pierce, Michael D. Green, Corey W. Speers

**Affiliations:** ^1^ Department of Radiation Oncology, University of Michigan, Ann Arbor, MI, United States; ^2^ Rogel Cancer Center, University of Michigan, Ann Arbor, MI, United States; ^3^ Department of Pharmacology, University of Michigan, Ann Arbor, MI, United States; ^4^ Graduate Program in Immunology, University of Michigan Medical School, Ann Arbor, MI, United States; ^5^ Department of Biology, Saint Mary’s College, Notre Dame, IN, United States; ^6^ Department of Microbiology and Immunology, University of Michigan, Ann Arbor, MI, United States; ^7^ Department of Radiation Oncology, Veterans Affairs Ann Arbor Healthcare System, Ann Arbor, MI, United States; ^8^ Department of Radiation Oncology, University Hospitals Cleveland Medical Center, Case Western Reserve University, Case Comprehensive Cancer Center, Cleveland, OH, United States

**Keywords:** immune checkpoint inhibitors (ICI), radiotherapy, breast cancer, tumor immunology, radiation biology, immunotherapy

## Abstract

Breast cancer is the most prevalent non-skin cancer diagnosed in females and developing novel therapeutic strategies to improve patient outcomes is crucial. The immune system plays an integral role in the body’s response to breast cancer and modulating this immune response through immunotherapy is a promising therapeutic option. Although immune checkpoint inhibitors were recently approved for the treatment of breast cancer patients, not all patients respond to immune checkpoint inhibitors as a monotherapy, highlighting the need to better understand the biology underlying patient response. Additionally, as radiotherapy is a critical component of breast cancer treatment, understanding the interplay of radiation and immune checkpoint inhibitors will be vital as recent studies suggest that combined therapies may induce synergistic effects in preclinical models of breast cancer. This review will discuss the mechanisms supporting combined approaches with radiotherapy and immune checkpoint inhibitors for the treatment of breast cancer. Moreover, this review will analyze the current clinical trials examining combined approaches of radiotherapy, immunotherapy, chemotherapy, and targeted therapy. Finally, this review will evaluate data regarding treatment tolerance and potential biomarkers for these emerging therapies aimed at improving breast cancer outcomes.

## Introduction

Breast cancer (BC) is the most common non-cutaneous malignancy diagnosed in females, accounting for nearly one-third of all new cancer diagnoses (1). During 2022, in the United States, approximately 287,850 females will be diagnosed with breast cancer, while over 43,000 females will ultimately succumb to their disease (1). Breast cancer incidence has increased in female patients, coinciding with an increase in obesity and decline in fertility rates (1, 2). Early detection and improved loco-regional and systemic therapies have led to improved outcomes among breast cancer patients in recent years (3). However, breast cancer is a heterogenous disease with diverse molecular subtypes, clinical classifications, and genetic variations (3, 4). Using the most common definition, breast cancer is divided into four molecular subtypes—luminal A, luminal B, HER2^+^, and triple negative breast cancer (TNBC)—based upon the presence or absence of important hormone receptors, including the estrogen receptor (ER), progesterone receptor (PR), and human epidermal growth factor receptor 2 (HER2) (4). This heterogeneity at the tumor level results in different responses to therapy (3–5). Importantly, TNBC is the most aggressive breast cancer subset that disproportionately impacts patients of color and younger patients (4, 6–8). Significantly, more effective therapies for TNBC are desperately needed.

Locally advanced breast cancer is treated via a trimodal approach that includes surgery, chemotherapy, and radiotherapy. Recent advances in precision medicine have been developed to target the molecular differences that exist in breast cancer (3). Endocrine therapies, including the selective estrogen receptor modulator (SERM) tamoxifen, selective estrogen degrader (SERD) fulvestrant, or the aromatase inhibitors anastrozole and exemestane, target the estrogen receptor found in ER^+^ breast cancer (9). Other precision medicine advancements used in the management of metastatic breast cancer include small molecule inhibitors of key modulators of breast cancer growth and survival. For example, inhibiting the cyclin dependent kinases 4 and 6 (CDK4/6) mechanistically prevents the progression of cancerous cells through the cell cycle, while inhibiting poly (adenosine diphosphate-ribose) polymerase (PARP) impairs DNA repair (10, 11). While these targeted therapies improve survival, therapeutic resistance is common, and the discovery of additional treatment options are warranted.

An emerging therapeutic option for treating breast cancer is immunotherapy, which enables a patient’s immune system to recognize and eliminate cancerous cells. Cancer cells evade the immune system by expressing immune checkpoints: inhibitory molecules that hinder the immune system’s ability to eliminate cancer. Immune checkpoint inhibitors (ICIs) block these immune checkpoints or “brakes” on the immune system, resulting in an increase in antitumor immunity and the eradication of cancerous cells. Currently, clinically utilized ICIs target the programmed death receptor 1 (PD-1)-programmed death ligand 1 (PD-L1) or cytotoxic T-lymphocyte-associated protein 4 (CTLA-4) axes (12). ICIs have been most clinically successful in the management of melanoma (13), non-small cell lung cancer (NSCLC) (14), and bladder cancer (15). Overall, more than 40% of all cancer patients are eligible to receive ICIs (16, 17). Importantly, recent studies suggest that ICIs are effective for the treatment of breast cancer patients, although it was originally believed that these patients would respond poorly to immunotherapies due to this disease being a relatively nonimmunogenic cancer (18). Of all breast cancer subtypes, immunotherapy is particularly promising for the treatment of TNBC that cannot be treated via hormone therapies due to not expressing commonly targeted hormone receptors—including the ER, PR, and HER2. Immunotherapy may also be promising for treating this subset of breast cancer, since treatment resistance to standard therapies—like chemotherapy and radiotherapy—remains a significant clinical issue for TNBC patients (19, 20).

Combining radiotherapy with immunotherapy for the treatment of aggressive breast cancers may improve treatment efficacy. Early preclinical studies demonstrate that radiotherapy promotes antigen presentation in tumor cells by causing DNA damage, altering transcription, and potentially leading to presentation of immunogenic peptides (21, 22). By promoting the presentation of immunogenic peptides, the recognition of cancer cells by T cells can be enhanced to reactivate the body against the tumor, thus, overcoming the immunosuppressive effects of immune checkpoints. Clinical studies assessing the effectiveness of multimodal approaches incorporating radiotherapy and immunotherapy in breast cancer are ongoing. While combining immunotherapy and radiotherapy to treat aggressive breast cancers is clinically promising, additional research is necessary to determine the mechanisms underlying this therapeutic approach. This review will cover the cellular and molecular regulators of antitumor immunity as well as review the preclinical and clinical advances supporting immunotherapy as a treatment option for breast cancer patients. Throughout this review, we place a special emphasis on emerging therapeutic approaches and clinical trials combining immunotherapy with radiotherapy to treat breast cancer.

### Immune microenvironment in breast cancer

The immune system is a powerful mediator in protecting the body against foreign pathogens, and importantly plays a crucial role in safeguarding the body from self-cells that become cancerous. Paradoxically, the immune system can play both supportive and inhibitory roles in breast cancer progression and is an important pharmacological target to improve patient outcomes (23). Tumors are classified based upon the presence and location of immune cells in the tumor microenvironment (TME), where noninflamed (“cold”) tumors have a low infiltration of lymphocytes and inflamed (“hot”) tumors have a high infiltration of lymphocytes (24). Noninflamed tumors can also have an absence of infiltrating lymphocytes or have lymphocytes only on the peripheral edges of the tumor (“excluded”) (25). Additionally, antitumor immunity is dependent on the immune tone of the TME, with both immunosuppressive and immunostimulatory milieu being common. This is relevant in breast cancer carcinogenesis, where both the innate and adaptive immune system contribute to cancer development and immune evasion (26).

Tumor-associated macrophages (TAMs) are innate immune cells found within the TME that have pro-tumorigenic and anti-tumorigenic effector mechanisms in the context of cancer (27). Macrophages are divided into M1-like macrophages that exert antitumor effects and M2-like macrophages that exert pro-tumorigenic effects; however, these phenotypes are plastic and can be pharmacologically reprogrammed (27). In breast cancer, it has been known for the past two decades that macrophages can promote malignant transformation (28), while monocyte-derived macrophages additionally contribute to breast cancer metastasis (29). FOLR2^+^ macrophages are a specific subset of TAMs enriched predominantly in healthy mammary glands that positively correlate with CD8^+^ T cells (30). Contrastingly, TREM2^+^ macrophages are a subset of TAMs expressed primarily in cancerous breast tissue that contribute to tumor development (30). Additionally, in both TNBC and hormone receptor-positive (HR^+^) breast cancer, CD163^+^ TAMs are derived from circulating monocytes and contribute to immunosuppression (31). Neutrophils, another innate cell lineage, can also exert multifaceted pro-tumorigenic and anti-tumorigenic effects under different contexts (32). Within TNBC, there are dichotomous neutrophil-enriched subtypes (NES) and macrophage-enriched subtypes (MES). Specifically, the NES subtype displays an abundance of immunosuppressive neutrophils and is resistant to ICIs, whereas the MES subtype demonstrates mixed responses to ICIs (33). Furthermore, myeloid-derived suppressor cells (MDSCs) are a heterogeneous population of immature myeloid cells of the innate immune system that suppress CD8^+^ T cells and other immune cells in the TME, promoting tumor progression (34). Elevated levels of circulating MDSCs were present more often in breast cancer patients than in healthy volunteers and were even higher in patients with metastatic disease (35). MDSC crosstalk signaling promotes breast cancer progression in part through the STAT3 and NOTCH signaling pathways (36). In all, these cells of the innate immune system exert multifaceted effects in the TME and execute significant roles in cancer progression and immune surveillance.

Tumor-infiltrating lymphocytes (TILs) collectively refer to the populations of lymphocytes in the tumor. This population of white blood cells includes T lymphocytes (T cells), B lymphocytes (B cells), and natural killer (NK) cells (37, 38). T cells compose approximately 75% of TILs and consist of different subsets including cytotoxic CD8^+^ T cells, CD4^+^ T cells, and regulatory T cells (Tregs) that all contribute to the adaptive immune response (38, 39). The presence of TILs is associated with improved disease outcomes in breast cancer patients (40, 41). CD8^+^ T cells are directly cytotoxic to tumor cells, while CD4^+^ T cells can promote antitumor immunity through the secretion of inflammatory cytokines (42). Meanwhile, some immune cell populations may induce immunosuppressive effects in the TME. For example, CD4^+^ Tregs restrain the activation and function of CD8^+^ T cells (43). While it is well-established that CD8^+^ TILs are a favorable prognostic indicator and positively correlate with relapse-free survival in breast cancer (44), the T cell subtypes present in breast cancer are not fully understood (45). CD8^+^ tissue-resident memory (T_RM_) cells are one subset of CD8^+^ TILs contributing to immunity that express cytotoxic molecules and immune checkpoint proteins (46). Interestingly, CD8^+^ TRM cells are associated with improved relapse-free survival (RFS) in TNBC cancer patients (45). In early-stage TNBC patients, the presence of T_RM_s is associated with improved patient outcomes—including increased survival and decreased rates of recurrence (46). Increased intra-tumoral expression of CD39^+^PD-1^+^CD8^+^ T cells, another subset of CD8^+^ TILs, correlates with longer disease-free survival in breast cancer patients (47). In breast cancer, FOXP3^+^ Tregs are a distinct population of T cells associated with more aggressive forms of breast cancer, including a higher risk of relapse and decrease in survival (48). Additionally, intratumoral Tregs from breast cancer tumors have increased expression of the chemokine receptor CCR8, suggesting a unique phenotype and function of these cells in human breast cancer patients (49). B lymphocytes are a humoral cell population of the adaptive immune system that can contribute to both antitumor immune responses and potentiate cancer development (50). B lymphocytes are less prevalent in invasive breast cancers in comparison to early ductal carcinoma *in situ* (50). Importantly, the presence of immune infiltrates in the breast tumor may correlate to patient response to therapy. In the SweBCG91RT trial, immune infiltrates, in the form of CD8^+^ T cells and FOXP3^+^ T cells, were examined in early-stage breast cancer patients that received breast-conserving surgery (BCS) and postoperative radiotherapy. In this trial, early-stage breast cancer patients with antitumoral immune infiltrates had a decreased risk of recurrence, while the addition of radiotherapy to these patients was found to have limited benefits (51). In summary, a variety of lymphocytes are present in breast tissue and many of these lymphocytes play dual roles in carcinogenesis and immune recognition.

Of the breast cancer subtypes, TNBC is associated with the highest lymphocyte infiltration, followed by HER2^+^ breast cancer, and finally by HR^+^, HER2^-^ breast cancer (41). Importantly, lymphocyte infiltration in breast cancer patients varies significantly from 1.1% to 44%, which is independent from tumor size (52). In a study that examined CD8^+^ T cell infiltration among 12,439 breast cancer patients, the presence of intratumoral CD8^+^ T cells was associated with a significant reduction in risk of death in both ER^-^ and ER^+^, HER2^+^ breast cancer. Specifically, intratumoral CD8^+^ T cell expression was associated with a 28% reduction in mortality for TNBC and HER2^+^ tumors and 27% reduction in mortality for ER^+^, HER2^+^ tumors (53). Furthermore, there have also been differences found in the tumor immune microenvironment of African American breast cancer patients compared to non-African American patients, which may be contributed to socioeconomic and ancestry factors. African American TNBC patients display an increase in gene expression of immune pathways and an increase in immune infiltration—providing rationale for the application of immunotherapies for these patient populations (54). Inflammatory breast cancer (IBC) is a rare type of breast cancer which clinically presents with distinct rapid and substantial inflammation of the breast (55). IBC has a unique tumor microenvironment composition compared to other breast cancers (56). Emerging evidence suggests that the tumor microenvironments of IBC tumors is associated with an increase in CD8^+^ T cell infiltration (57, 58) and tumor-associated macrophages (59, 60); however, the effects of the immune system and underlying molecular pathways of IBC carcinogenesis are not fully defined (61). In summary, more research is necessary to understand the implications of immunotherapy for other breast cancer subsets, including HR^+^ breast cancers and IBC.

### Regulators of immune responses in breast cancer

The immunogenicity of tumors is influenced by multiple factors, including the mutational load of the tumor. Cancerous cells accumulate variable levels of somatic mutations, which may result in the production of neoantigens and tumor-specific antigens (TSAs) (62–64). These antigens are recognized by the immune system to distinguish cancer cells from healthy, noncancerous cells (62). The ability of cytotoxic CD8^+^ T cells to recognize neoantigens produced by tumor cells was reported in the early 1990s and provided an important insight into the antitumor effects of T cells in cancer (65, 66). Cancer immunotherapies are often developed to target these neoantigens because they are tumor-specific and, thus, an attractive target for minimizing on-target, off-tumor effects (63, 67). Compared to other malignancies, breast cancer has less than the median number of somatic mutations (64). Only 5% of all breast cancers are hypermutated and carry a significant load of somatic mutations. Additionally, in breast cancer, the APOBEC signature, a signature that represents dysregulated AID/APOBEC cytidine deaminases, is the primary mutational process leading to these hypermutations (68). As tumor mutational load correlates with response to immunotherapy, from the perspective of antigen presentation, breast cancer is deemed relatively non-immunogenic.

Disruption and dysregulation of the cancer immunity cycle promotes carcinogenesis. Data from The Cancer Genome Atlas (TCGA) and Molecular Taxonomy of Breast Cancer International Consortium (METABRIC) breast cancer cohorts suggest that malfunction of the cancer immunity cycle contributes to disease progression and serves as a prognostic biomarker (69). Avoiding immune clearance is an important hallmark of cancer that enables cancer cells to expand independently from the inhibitory effects of the immune system (65, 70). In the cancer immunity cycle, antigens produced by cancer cells are sampled by antigen-presenting cells (APCs) such as macrophages, dendritic cells, and B cells (65). APCs then present the antigens via major histocompatibility complexes I or II (MHCI/II) (65). Naïve T cells can recognize these antigens when their T cell receptor (TCR) binds to the MHC on the APC, and this interaction is stabilized by the co-receptors CD4 or CD8. This TCR recognition of the peptide-MHC complex is insufficient to fully activate T cells. An additional co-stimulation signal is required, which occurs when costimulatory molecules, such as CD28, on the T cell recognize signals, such as CD80/86, on the APC. Following these two signals, the APC will release cytokines, such as IL-2, to further direct the activation and differentiation of T cells. Once activated, T cells egress from the lymph nodes, traffic through the blood, and enter the TME (65). Trafficked T cells may then utilize their tumor antigen-specific TCRs to bind to neoantigens presented on MHC-I by the cancer cell, allowing for granzyme and perforin driven cytotoxicity. The overall effect of this pathway is dependent on which population of T cells is recruited to the tumor microenvironment.

In breast cancer, there are several mechanisms utilized by cancer cells to avoid recognition by the cancer immunity cycle (71). One way tumor cells can avoid immune recognition is via loss of MHC class I antigen presentation, which prevents the tumor cells from being recognized by CD8^+^ T cells (72). In breast cancer cells, this may occur in part through the protein MAL2 that promotes endocytosis of tumor antigens (73). Moreover, breast cancer cells can deplete the costimulatory receptor needed for T cell activation when CTLA-4 on tumor cells and CD80 on APCs promote trans-endocytosis of CD80 (74). Furthermore, by expressing immune checkpoints, cancer cells can target and inhibit the effector functions of T cells, including suppression of antitumor cytokine secretion and T cell proliferation (71). Collectively, these studies illustrate the many ways that breast cancer can avoid recognition by the cancer immunity cycle.

**Figure 1:**
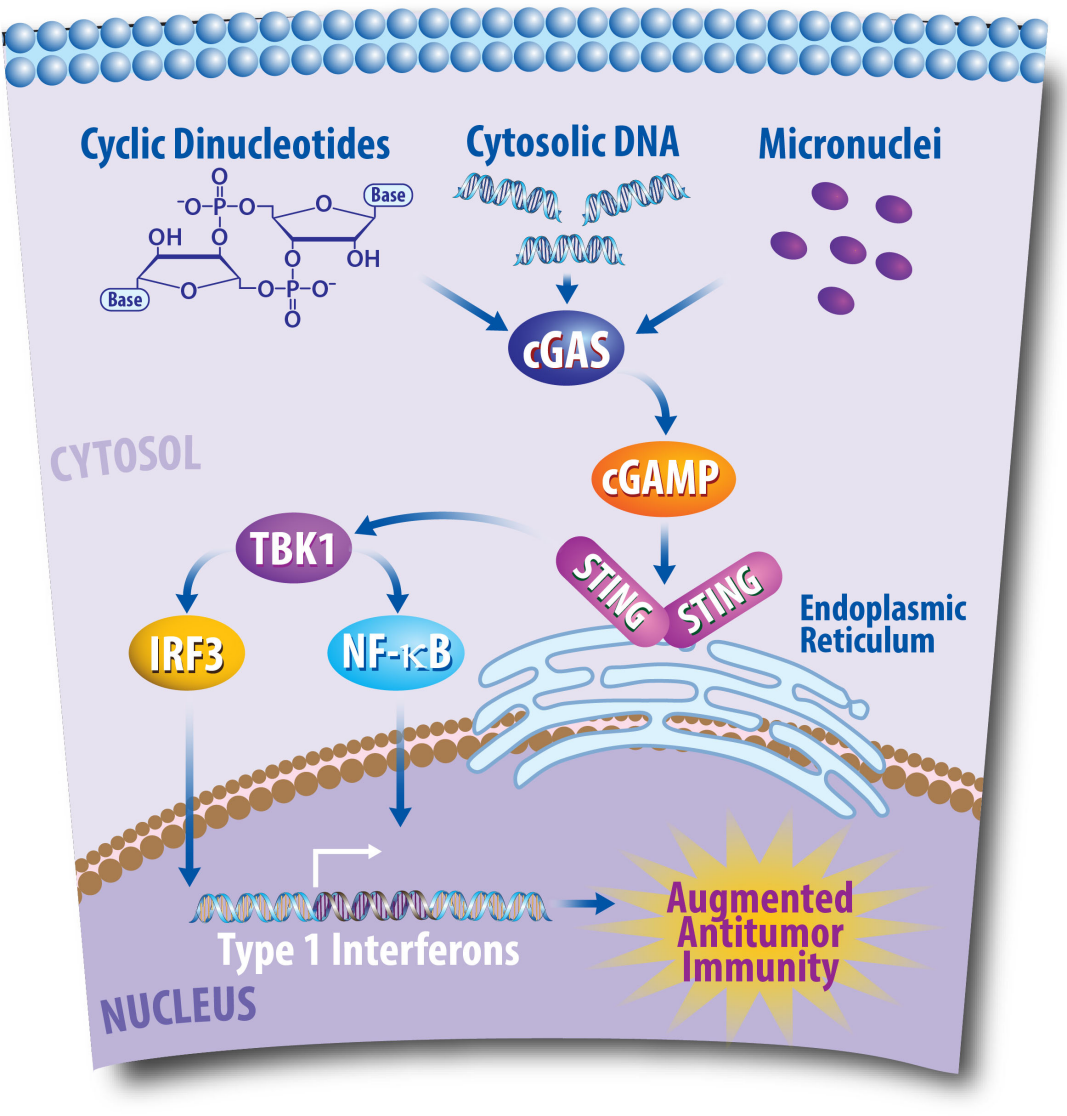
The Cyclic GMP-AMP Synthase-Stimulator of Interferon Genes (cGAS/STING) Pathway Plays a Critical Role in Antitumor Immunity. Following DNA damaging events, DNA fragments enter the cytoplasm of cancer cells. This cytosolic DNA is then recognized by the cytoplasmic sensor cGAS, which can then produce cyclic GMP-AMP (cGAMP). Consequently, cGAMP promotes the recruitment of STING molecules in the endoplasmic reticulum, which leads to TANK-binding kinase 1 (TBK1) phosphorylating interferon regulatory factor 3 (IRF3), and nuclear factor-κB (NF- κB). IRF3 and NF- κB then translocate to the nucleus to promote transcription of type I interferons, which can lead to an antitumor response via the promotion of T cell infiltration into the tumor microenvironment.

An immune pathway especially critical for modulating immune responses to cancer is the cyclic GMP-AMP synthase-stimulator of interferon genes (cGAS/STING) pathway, as represented in **Figure 1** (75). The stimulator of interferon genes (STING) is an endoplasmic reticulum (EnR)-bound, transmembrane protein that stimulates the transcription of numerous immune pathways following the recognition of cyclic dinucleotides (CDNs) and cytosolic DNA (cDNA) (75–77). CDNs and cDNA can be produced from viruses, bacteria, and diseased states including cancer (76). These cytoplasmic molecules of genetic information are consequently recognized by cyclic GMP-AMP synthase (cGAS), which produces cyclic GMP-AMP (cGAMP) (76, 77). Chromosomal instability (CIN)—another hallmark of cancer—occurs following chromosomal segregation errors during mitosis and can also activate the cGAS/STING pathway in cancer cells (70, 78). Moreover, in addition to promoting an antitumor immune response through the cGAS/STING pathway, CIN can also promote the activation of other immune cells, including natural killer cells to promote antitumor immunity (79). Micronuclei formation can additionally promote the cGAS/STING pathway to activate an immune response (80). Production of cGAMP by such means activates STING via binding with two STING molecules in the EnR, which leads to STING interacting with TANK-binding kinase 1 (TBK1) (76, 77). TBK1 can then phosphorylate type 1 interferon (T1IFN) transcription factors including interferon regulatory factor 3 (IRF3) and nuclear factor-κB (NF-κB) that promote gene transcription after translocation to the nucleus (76, 77). The cGAS/STING pathway and activation of T1IFNs also plays critical roles in cancer (81). For example, T1IFN production is often associated with T cell infiltration that promotes immune responses against tumors (76, 82, 83). In breast cancer, perinuclear expression of STING was recently found to be associated with improved prognosis in ER^+^ breast cancers (84). Consequently, the development of STING agonists has been explored as a therapy for the treatment of breast cancer to induce an antitumor response and improve the efficacy of additional immunotherapeutic approaches (85, 86). In short, the cGAS/STING pathway plays a critical role in cancer and is a potential pharmacological target for treating cancer patients.

### The role of immunotherapy in breast cancer

Immunotherapeutic approaches aimed at improving cancer control rates in breast cancer patients include cancer vaccines, adoptive cell transfer, and ICIs (87, 88). Cancer vaccines target distinct antigens upregulated in the tumors of cancer patients and provide immunological memory (89). Mechanistically, cancer vaccines seek to trigger an immune response via machinery that promotes the presentation of tumor antigens to the immune system and via adjuvants that cause a proinflammatory response to activate the immune system (89). Current research is focused on developing vaccines that can prevent the progression of aggressive breast cancers—such as triple negative disease (NCT04674306)—and combining breast cancer vaccines with other treatment approaches (NCT00082641, NCT03789097). For instance, mRNA vaccines have recently been successful in the context of COVID-19 and are currently being explored for use in breast cancer (90). Significant work has been done to study the efficacy of breast cancer vaccines both preclinically and clinically; however, most studies have failed to produce significant responses in patients, which may be attributed to the heterogeneity of breast cancer (89, 91).

ICIs have revolutionized cancer therapeutics, leading to Dr. James P. Allison and Dr. Tasuku Honjo being awarded the Nobel Prize in Physiology or Medicine in 2018 (92). One class of ICIs target programmed death-ligand 1 (PD-L1 or B7-H1), which serves to inhibit the immune system by binding to PD-1 on T cells and dampening their cytotoxic abilities (93). PD-L1 is expressed on a myriad of immune cells, including antigen presenting cells, T cells, and B cells, and interacts with its receptor, PD-1, expressed on T cells (94, 95). Mechanistically, PD-L1 and PD-1 interactions suppress tumor immunity by causing T cell apoptosis, anergy, exhaustion, and IL-10 expression (94). Expression of PD-L1 and PD-1 in the tumor microenvironment is a common cancer immune evasion strategy (94). Cytotoxic T-lymphocyte-associated protein 4 (CTLA-4 or CD152) is another immune checkpoint receptor expressed on T cells that has a high affinity for CD80 and CD86, which are necessary for T cell co-stimulation (96, 97). CTLA-4 outcompetes the co-stimulatory molecule CD28 to induce immune suppression (97, 98). In breast cancer, TCGA analyses suggest that TNBC patients express higher levels of PD-L1 as compared to patients with other breast cancer subtypes with approximately 20% of TNBC samples expressing significant levels of PD-L1 (99). While PD(L)-1 inhibition is clinically efficacious in many cancer types, PD-L1 expression poorly predicts clinical benefit, emphasizing the demand for clinical trials evaluating efficacy as well as the need for better biomarkers of treatment response (100).

Importantly, clinical trials have tested the efficacy of ICIs in TNBC. The Phase Ib KEYNOTE-012 clinical trial (NCT0184883) tested whether pembrolizumab (anti-PD-1) was tolerable in patients with PD-L1^+^ advanced TNBC. This study found that pembrolizumab had an acceptable safety profile, with an overall response rate of 18.5% (101). In the Phase II KEYNOTE-086 trial (NCT02447003), 254 female patients with metastatic TNBC received pembrolizumab in either the second line setting or the first line setting. In the second line setting, patients unselected for PD-L1 expression had an objective response rate (ORR) of 5.3%, while in the first line setting, PD-L1^+^ patients had an ORR of 21.4%. Tolerability was reaffirmed in both cohorts (102, 103). This trial led to the randomized, open-label Phase III KEYNOTE-119 (NCT02555657) trial that examined the efficacy of pembrolizumab versus single agent chemotherapy in patients with PD-L^+^ metastatic TNBC. In this trial, PD-L1^+^ status was characterized by patient PD-L1 combined positive scores (CPS), defined as the ratio of PD-L1^+^ tumor cells, lymphocytes, and macrophages out of total tumor cells multiplied by 100. Pembrolizumab improved the median overall survival (OS) from 11.6 months to 12.7 months as compared to chemotherapy in patients with a CPS of 10 or higher (104). KEYNOTE-119 motivated the Phase III, double-blind, randomized trials KEYNOTE-355 (NCT02819518) and KEYNOTE-522 (NCT03036488) (105, 106). In KEYNOTE-355, 847 patients with metastatic TNBC or previously untreated, locally recurrent inoperable breast cancer were randomized 2:1 to pembrolizumab and chemotherapy (specifically, paclitaxel, nab-paclitaxel, or gemcitabine plus carboplatin) or placebo and chemotherapy. The co-primary endpoints of this trial were overall survival and progression-free survival, and patients were stratified by PD-L1 expression. Pembrolizumab and chemotherapy improved the median progression-free survival from 5.6 months to 9.7 months for patients with high PD-L1^+^ scores, providing the clinical rationale for using this combined therapy as a first-line treatment for metastatic TNBC (105). Furthermore, recent data supports that in patients with advanced TNBC with a CPS of 10 or more, the median overall survival increased from 16.1 months in the placebo-chemotherapy group to 23.0 months in the pembrolizumab-chemotherapy group. Similarly, in patients with a CPS of 1 or more, the median overall survival increased from 16 months in the placebo-chemotherapy group to 17.6 months in the pembrolizumab-chemotherapy group (107). In KEYNOTE-522, 1,174 patients with either previously untreated stage II breast cancer or stage III TNBC were randomly assigned 2:1 to receive neoadjuvant and adjuvant pembrolizumab with chemotherapy (either carboplatin or paclitaxel) or placebo with chemotherapy. All patients also received standard of care neoadjuvant doxorubicin–cyclophosphamide or epirubicin–cyclophosphamide. KEYNOTE-522 had two primary endpoints of pathological complete response (pCR, defined as the absence of invasive disease) and event-free survival. Pembrolizumab and chemotherapy significantly increased the pCR compared to chemotherapy alone (51.2% to 64.8%), and these data were foundational to the FDA-approval for pembrolizumab use in combination with chemotherapy for this patient population (106). Thus, these trials have established pembrolizumab as an important treatment for both metastatic and non-metastatic TNBC. Additionally, preliminary data suggests atezolizumab, a humanized anti-PD-L1 IgG1 antibody, is active in PD-L1^+^ locally advanced or metastatic TNBC; however, accelerated approval was later rescinded based on subsequent demonstration of limited clinical efficacy (108–110).

Clinical trials have also assessed the efficacy of ICIs in the management of HR^+^ breast cancers. In the Phase 1b KEYNOTE-028 study, patients with ER^+^, HER2^-^ breast cancer with PD-L1^+^ tumors received pembrolizumab and achieved an ORR of 12% (NCT02054806) (111). Furthermore, in the Phase 1b JAVELIN study, which tested the safety of avelumab, 43% of patients had HR^+^, HER2^-^ breast cancer and the ORR was 3% (NCT01772004) (112). The combination of pembrolizumab with chemotherapy (113) and cyclin-dependent kinase inhibitors (114) in this patient population has also not led to improvements in clinical outcomes. These trials highlight that ICIs have limited clinical activity in HR^+^ breast cancer. The poor efficacy of ICIs for the treatment of HR^+^ breast cancer may be, in part, due to the limited immune cell infiltrate in these tumors (115). The effects of ICIs are also currently being examined for the treatment of inflammatory breast cancer (116). A Phase II study (NCT02411656) is currently assessing the effects of pembrolizumab in metastatic or recurrent inflammatory breast cancer patients. Moreover, a Phase II study is currently examining the effect of pembrolizumab in combination with hormone therapy during or after radiotherapy for patients with HR^+^ inflammatory breast cancer who did not respond to neoadjuvant chemotherapy alone (NCT02971748). Clinical trials are currently recruiting patients to assess the effect of ICIs in combination with chemotherapy (NCT03515798, NCT05093387) for the treatment of inflammatory breast cancer. Furthermore, a recent case study suggests clinical promise in combining pembrolizumab and chemotherapy for treating inflammatory breast cancer (117), while additional studies are underway to identify novel biomarkers for anti-PD-1 therapy in this disease, including peripheral T cell exhaustion and clonality markers (118). Moreover, beyond the scope of immunotherapy, current clinical trials are also examining combined therapies of radiotherapy and PARP inhibition for the treatment of inflammatory breast cancer (NCT03598257).

Adoptive cell transfer (ACT) therapy functions by transferring immune cells into cancer patients. Chimeric antigen receptor (CAR)-T cells enable improved T cell recognition of cancers via bypass of the common cancer immune evasion strategies of MHC downregulation and co-stimulation blockade (119). CAR-T cells are composed of single-chain variable fragments (scFv) fused to a costimulatory molecule which is fused to the intracellular CD3ζ signaling domain. The scFv recognizes antigen expressed on the surface of tumor cells. The CD3ζ immunotyrosine activation motif (ITAM) generates T cell activation signal 1 and the intracellular costimulatory domain generates signal 2. This allows CAR-T cells to become fully activated following recognition of peptide without the need for MHC presentation or additional co-stimulation. CAR-T cells, are engineered for each individual patient by first collecting T cells from the peripheral blood of cancer patients, transducing them *ex vivo* to express the appropriate CAR, expanding, and validating these CAR-T cells, and then reintroducing these cells into patients (120). CAR-T cell therapies are a powerful tool for treating cancer patients in that these modified cells can also persist in patients for extended periods, providing significant support to the immune systems of patients undergoing CAR-T cell therapy (119). Currently, there are six CAR-T cell therapies approved for clinical use in hematologic malignancies (121). However, there are no CARs currently approved for use in breast cancer. In developing CAR-T cell therapies, it is important that the antigens being targeted are enriched in the tumor and not the healthy tissues of patients to prevent “on-target off-tumor” adverse events (119, 120). Additionally, CARs are limited in that they can only be directed towards surface-expressed antigens. CAR-T cells have shown limited promise in solid tumors due to a variety of challenges, including poor T cell infiltration into tumors and immunosuppressive tumor microenvironments, although there is significant work underway to overcome these obstacles. For the treatment of breast cancer, preclinical studies are ongoing to examine the effects of CAR-T cell therapy on various tumor specific antigens including mucin 1 (MUC1), HER2, Lewis Y, mesothelin, and folate receptor alpha (FR-α) (119). Clinical trials are underway to assess the effects of CAR-T cell therapy for treating breast cancer, including CAR-T cells recognizing epithelial cell adhesion molecule (EpCAM) (NCT02915445), cleaved MUC1 (NCT04020575, NCT02792114), and ROR1 (NCT05274451). In addition to CAR-T cell therapy, tumor-infiltrating lymphocytes (TILs) are being examined as a type of adoptive cell transfer for the treatment of breast cancer. TIL therapy involves isolating tumor-infiltrating lymphocytes from patients, expanding them *ex vivo* with large amounts of IL-2 and other cytokines, then re-infusing them into the patient (122). Importantly, TIL therapy does not significantly modify the lymphocytes, and, unlike CAR-T therapy, TIL therapy assumes patient lymphocytes are able to recognize tumor neoantigens that exist in small quantities. Whole exome sequencing of breast cancer tissues revealed TNBC expresses more neoantigens than non-TNBC, suggesting TNBC patients may be good candidates for TIL therapy (123). An ongoing clinical trial (NCT01174121) seeks to use TIL therapy in metastatic breast cancer, and preliminary data has shown tumor regression in a subset of patients (124). Collectively, these studies suggest the importance of ACT therapies as a potential therapeutic approach for breast cancer.

Despite promise of these therapies as single-agent therapies, additional studies are underway to find ways to increase patient responses to ACT by combining with radiotherapy or other forms of immunotherapy. For example, studies are currently examining combining radiotherapy with CAR-T cell therapy as a means to improve patient response to adoptive T cell transfer and overcome resistance in solid tumors (125). The effect of CAR-T cell therapy and internal radiotherapy are beginning to be evaluated for the treatment of liver metastases in breast cancer patients in a Phase 1b trial (NCT02416466), and results demonstrated some efficacy of the combination therapy with minimal toxicities (126). Moreover, a study recently examined the impact of combining infusion of *ex vivo* expanded NK cells into a human TNBC xenograft model with radiotherapy and found that the combination therapy significantly decreased primary tumor growth while minimizing toxicity (127). Combining CAR-T cell therapy with anti-PD-1 led to reduced tumor weight and improved CAR-T cell infiltration into the TME in a murine breast cancer model, demonstrating this combination therapy strategy may also be promising for treating breast cancer patients (128). While adoptive cell transfer strategies have shown some promise in the treatment of breast cancer in preclinical models, there has yet to be significant clinical efficacy in these solid malignancies.

In addition to immunotherapy, monoclonal antibodies (mABs) directed either towards tumor-specific antigens or mediators of oncogenic signaling have been used in breast cancer for more than twenty years. Monoclonal antibodies that target growth signaling can prevent cancer cell proliferation and ultimately lead to apoptosis. Additionally, these monoclonal antibodies can mediate antibody-dependent cellular cytotoxicity (ADCC), engaging the immune system to recognize cancer cells coated with antibodies bound to the surface of the cell (129). Trastuzumab is a clinically approved anti-HER2 mAb which improves the overall survival of patients with HER2^+^ breast cancers (130). Pertuzumab targets a distinct epitope of HER2 and is another mAB used in the management of HER2^+^ breast cancer. Consequently, mABs are a promising immunotherapy strategy for the treatment of breast cancer patients; however, these therapies are not efficacious for the treatment of triple negative disease that does not express the HER2 receptor. Interestingly, even in HER2-low expression tumors, the DESTINY-Breast04 trial recently demonstrated improved survival in women with metastatic HER2-low expressing tumors using the HER2 targeted therapy trastuzumab deruxtecan (131). Whether HER2-targeted therapies combined with ICIs will be even more effective remains an area of active clinical interest.

### The impacts of immunotherapy and radiotherapy in breast cancer

Unfortunately, only 10% of patients with TNBC respond to immune checkpoint inhibitor monotherapy (85). Thus, there is an unmet need to develop more effective therapeutic strategies to improve patient responses to ICIs. One strategy to improve therapeutic efficacy of ICIs may be to combine immunotherapy with other effective breast cancer treatment modalities such as radiotherapy. For this review, we will primarily focus on combined approaches with immunotherapy—in the form of ICIs—and radiotherapy. However, other reviews have examined the effects of combining radiotherapy with cancer vaccines (132, 133), anti-HER2 therapies (134), or CAR-T cell therapy (135).

Radiotherapy is a mainstay breast cancer therapy first used to treat breast cancer patients in as early as the 1800s (136, 137). Clinical radiotherapy involves the delivery of fractionated doses of ionizing radiation to the affected cancerous breast tissue while sparing the surrounding benign tissues. This results in targeted disruption of tumor cells through induction of DNA damage, alterations in the cell cycle, and ultimately cancer cell death (138–140). Multiple randomized clinical trials have effectively established that radiotherapy reduces local recurrence in both invasive and noninvasive breast cancers, in addition to reducing the risk of breast cancer death (141–143). Specifically, after breast-conserving therapy, radiotherapy reduced the 10-year risk of a local or distant recurrence from 35.0% to 19.3% and reduced the 15-year breast cancer death risk from 25.2% to 21.4% (141). Despite such benefits, radiotherapy can have pleotropic effects on the immune system. For instance, large field and total body irradiation, which is clinically indicated in the management of hematologic malignancies (144), is used to induce profound lymphopenia. Meanwhile, localized radiotherapy may promote antitumor immune responses. An early study in the 1950s first described a phenomenon known as the “abscopal effect” that showed a correlation between the immune system and localized radiotherapy (145). The abscopal effect (in Latin, *ab*: away from, *scopus*: target) postulates that radiotherapy delivered to one part of the body can reduce tumor size systemically, in regions outside of where radiation is delivered (145–147). Literature suggests that this phenomenon occurs in part through the immune system (148–150), and immunotherapy is believed to promote abscopal effects (151). However, studies show that the abscopal effect is rare (146, 152) and unlikely to be broadly applicable clinically. An additional hallmark study of the late 1970s further expanded upon the connections between radiotherapy and the immune system to show that the efficacy of RT is dependent upon the immune system (153). Significantly, radiotherapy and immunotherapy provide synergistic tumor control when combined in preclinical models (154, 155). In fact, radiotherapy can sensitize even poorly immunogenic cancers including pancreatic cancer (156), head and neck squamous cell carcinoma (157), and breast cancer (158) to ICIs—which emphasizes the promise of combined radiotherapy and immunotherapy treatment modalities.

Notably, the effects of combination radiotherapy with ICIs in breast cancer models have been explored. A crucial study by Demaria et al. in 2005 illustrated the effects of combined radiotherapy and immune checkpoint inhibition in murine models of breast cancer (159). Specifically, combined local radiation with anti-CTLA-4 immune checkpoint inhibition in a poorly immunogenic murine breast cancer model resulted in prolonged survival and decreased lung metastases (159). Furthermore, later studies suggest that fractionated radiotherapy—as opposed to single-dose radiotherapy—induces systemic antitumor effects in combination with anti-CTLA-4 treatment in murine breast cancer models (160). These studies mutually suggest that radiotherapy combined with anti-CTLA-4 therapy promotes antitumor immunity in preclinical breast cancer models—providing rationale for combined use in the clinic (159, 160). Studies suggest that these effects of combined therapy depend on the immune cells present. In fact, in murine breast cancer models, the effects of radiotherapy and anti-CTLA-4 immunotherapy are dependent upon the presence of invariant natural killer T cells (161). Radiotherapy has also been found to induce CXCL16 release by breast cancer cells to attract effector T cells in murine models (162). Moreover, it has been proposed that the synergistic effects of radiotherapy and immune checkpoint inhibitors depend upon MTOR signaling (163) and tumor heterogeneity (164) in murine breast syngeneic models. While these studies display the synergistic effects of combined radiotherapy and ICIs for the treatment of breast cancer, more research is warranted to further understand the implications of these combined therapies.

Radiotherapy has been found to improve innate antitumor responses, deplete immunosuppressive cell types, and augment adaptive immune responses in combination with PD-1 blockade (165). Functionally, it is believed that radiotherapy activates the innate immune system via a process known as cross priming (166). As radiotherapy induces tumor cell death, these cells release neoantigens (167) that may be phagocytosed by nearby APCs. APCs can then activate the adaptive immune system, specifically CD8^+^ effector T cells, to kill cancer cells (166, 168). Consequently, the efficacy of radiotherapy specifically depends upon the presence of these cytotoxic cells (169). Interestingly, combining radiotherapy with immunotherapy has also been shown to jointly promote tumoral lipid oxidation-dependent ferroptosis via SLC7A11 (170). Radiotherapy can further induce the DNA damage response often associated with the synergistic effects of radiotherapy and immunotherapy. Targeting ataxia telangiectasia mutated (ATM)—a kinase that plays a role in the DNA damage response to double stranded DNA breaks induced by radiotherapy—sensitizes pancreatic cancer to ICIs, providing a mechanistic link for this observed synergy (171). Additionally, inhibition of DNA-dependent protein kinase (DNA-PK) has been shown to synergize with radiotherapy and modulate the immune system in pancreatic cancer models by increasing cytosolic double-stranded DNA and type 1 interferon signaling. Moreover, combined anti-PD-L1 with radiotherapy and DNA-PK inhibition further potentiates antitumoral immunity in preclinical pancreatic cancer models (172). These studies emphasize the complexity underlying the synergistic effects of combined radiotherapy and immunotherapy and can importantly be extended into the breast cancer space to determine the underlying mechanisms of such approaches.

**Figure 2:**
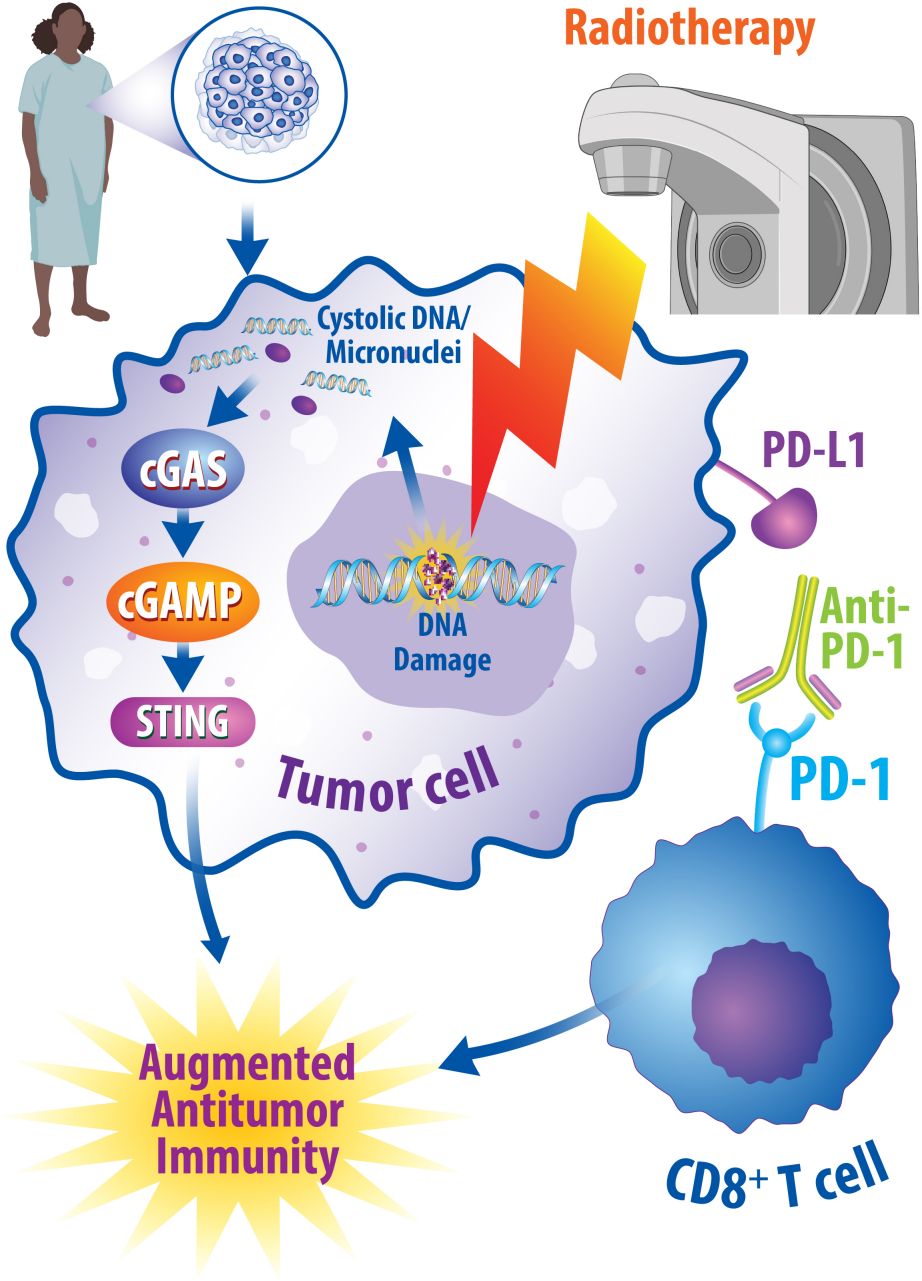
Radiotherapy and Immunotherapy Synergistically Promote Antitumor Immune Responses. One potential combined therapeutic approach is to combine radiotherapy with immune checkpoint inhibition. Radiotherapy promotes DNA damage within cancerous cells, which can consequently be recognized by cGAS and lead to activation of the cGAS/STING pathway to promote antitumor immunity through interferon signaling. Likewise, immune checkpoint inhibitors, such as anti-PD-1 monoclonal antibodies, can modulate an augmented antitumor immune response by turning off immune checkpoints. Under normal conditions, these checkpoints result in a decrease in the cytotoxic abilities of T cells; however, when turned off, this enhances the cytotoxic effects of T cells and results in enhanced antitumoral effects. Numerous preclinical and clinical studies suggest synergy exists in combining radiotherapy and immune checkpoint inhibitors in breast cancer patients and studies are currently underway to determine the best ways oncologists can implement these interactions.

While the precise mechanisms underlying the synergistic effects of radiotherapy and immunotherapy are not well established, studies have suggested that the cGAS/STING pathway may contribute to these combined effects as summarized in **Figure 2**. As discussed above, the cGAS/STING pathway plays a critical role in the antitumoral immune response by inducing interferon signaling following the recognition of cytosolic DNA (76). It is also well established that radiotherapy induces the cGAS/STING pathway to activate interferon signaling (173, 174). Importantly, interferon signaling can promote antitumor T cell responses (76, 81). It was also recently discovered that STING regulates radiotherapy sensitivity *in vivo* in part through the production of reactive oxygen species (ROS) (175). In human breast cancer cell lines and murine breast cancer models, inhibition of ectonucleotide pyrophosphatase phosphodiesterase 1 (ENPP1), a hydrolase of cGAMP, was recently found to increase extracellular cGAMP levels and synergize with radiotherapy to prevent tumor growth. The radiotherapy-induced increased production of extracellular cGAMP was subsequently sensed by STING and promoted the infiltration of dendritic cells and cytotoxic T cells into the tumor. Furthermore, depletion of extracellular cGAMP abrogated this immune cell infiltration in breast cancer models, suggesting that these radiation-induced immune effects are dependent upon the presence of extracellular cGAMP and the cGAS/STING pathway (176). Mechanistically, in human breast cancer cell lines, it has also been shown that the cGAS/STING pathway is required for interferon activation induced by combined radiotherapy and anti-CTLA-4 immune checkpoint inhibition (177). In addition to studying the effects of combined radiotherapy with anti-CTLA-4 treatments, preclinical studies suggest that radiotherapy and anti-PD-1/L1 therapy synergistically potentiate antitumor immunity in murine breast cancer models (178–180). Specifically, this antitumor immunity occurs in the form of reduced accumulation of myeloid-derived suppressor cells in the tumor (178), promotion of CD8^+^ T cell expansion (179), expansion of antigen-specific T cell responses (180), and reduction in tumor growth in non-irradiated tumor sites (181). Importantly, additional work is required to understand the contribution of other innate immune sensors and immune signaling pathways governing the synergistic interactions between radiotherapy and immunotherapy in breast cancer.

STING-dependent cytosolic sensing of DNA has been found to contribute to innate immunostimulatory responses following radiotherapy (173). However, there are also other pathways that link DNA damage to innate immune signaling. Nucleic acids can also be sensed by retinoic acid inducible gene-I (RIG-I)-like receptors (RLRs), Nod-like receptors (NLRs), and Toll-like receptors (TLRs) (182). Furthermore, the recognition of cytosolic DNA following viral infection has been found to activate a type I interferon response independently from toll-like receptors—further adding to the complexity of such pathways (183). When RIG-1 engages single and double stranded RNA, RIG-I complexes with mitochondrial antiviral-signaling protein (MAVS) and activates the TBK1 complex which ultimately promotes interferon signaling (184). In breast cancer, RIG-I agonists have been found to induce inflammatory transcription factors, type I interferons, and lymphocyte-recruiting chemokines (185).

The DHA-dependent protein kinase (DNA-PK) which, is required for nonhomologous end joining (NHEJ), also serves as another STING-independent innate immune sensor. DNA-PK can be activated by viral DNA leading to IRF3 and IRF7 dependent innate immune sensing (186). Interestingly, inhibition of DNA-PK has also been shown to augment radiation-induced interferon signaling in an RNA Polymerase III, Rig-I, and MAVS dependent fashion (172). TLRs have also been found to contribute to innate immune signaling in breast cancer (187). Specifically, Toll-like Receptor 9 (TLR9) can detect DNA released by tumor cells following chemotherapy leading to enhanced antigen presentation and improved antitumor immune responses (188). Consequently, TLR9 agonists have been examined as potential cancer therapeutics delivered in combination with other therapies (189). Combined TLR9 agonism and radiotherapy promotes systemic antitumor immunity in models of metastatic lung cancer and colon cancer (190). In a preclinical breast cancer mouse model resistant to PD-1, TLR9 agonists increased infiltration of CD8^+^ T cells into tumors and promoted IFN signaling (191). Collectively, these studies articulate the breadth of the pathways linking DNA damage and innate immune signaling.

While preclinical studies have illustrated the importance of combining radiotherapy with immunotherapy, clinical trials are also underway to assess these combined approaches. The single-arm Phase II clinical trial (NCT02730130) assessed the combination of pembrolizumab and radiotherapy in patients with metastatic TNBC and observed a 17.6% overall response rate, with minor adverse events as a result of combined therapy (192). In this study, radiotherapy was delivered at 30 Gy at five daily fractions to both PD-L1^+^ and PD-L1^-^ patients. Of the 9 patients observed through this trial, 3 patients with baseline PD-L1^+^ expression received a complete, durable response, which was similar to responses in studies where all patients had PD-L1^+^ metastatic TNBC (192). Phase II trials have also evaluated the combination of pembrolizumab and radiotherapy in patients with HR^+^, HER2^-^ heavily pretreated metastatic breast cancer (NCT03051672). This trial observed that pembrolizumab delivered prior to palliative radiotherapy (20 Gy in 5 fractions) did not result in any objective responses (193). These studies suggest that combined radiotherapy and immunotherapy may be more efficacious for patients with triple negative disease as opposed to HR^+^ breast cancers; however, additional research is necessary to fully determine the mechanisms of resistance in luminal breast cancer to immunotherapy.

Clinical trials are underway to study the effects of radiotherapy and ICIs in patients with breast cancer. These trials are summarized in **Table 1**. In addition to examining the effects of combined ICIs with radiotherapy in metastatic TNBC as discussed above (NCT02730130), such clinical trials are also examining combined therapies in metastatic HR^+^ breast cancer (NCT04756505). Importantly, many clinical trials are aimed at determining the survival outcome of combined therapies, as well as understanding the immune-enhancing effects of radiotherapy and immunotherapy in breast cancer patients. For example, preoperative delivery of radiation boost is being examined in combination with ICIs to enhance ICI efficacy in operable breast cancer (NCT04454528) and in TNBC and HR^+^/HER2^-^ breast tumors (NCT03366844) (194). Another study is assessing the effects of ICIs on the tumor microenvironment of TNBC patients prior to intraoperative radiotherapy (IORT) (NCT02977468). Trials are also examining the effects of novel therapeutic immune agents, including an antagonistic OX40 monoclonal antibody (NCT01862900) and the STING agonist TAK-676 (NCT04879849) combined with radiotherapy for the treatment of breast cancer patients. While many studies are examining the effects of the ICI pembrolizumab, studies are also examining the effects of the ICI nivolumab in combination with radiotherapy for the treatment of metastatic breast cancer brain metastases (NCT03807765) and patients with TNBC (NCT03818685). Together, these studies will help understand the effects of combined radiotherapy and ICIs in breast cancer patients and provide clinical rationale for combining these therapeutics with other available therapies such as chemotherapy.

**Table 1:** Trials examining the effects of combined radiotherapy and immune checkpoint inhibitors.

ClinicalTrials.gov identifier	Study title	Conditions	Therapeutic agent(s)	Radiotherapy	Phase and patients	Status (at time of publication)
**NCT02730130**	A Multicenter Single Arm Phase II Study to Assess the Efficacy of Pembrolizumab Plus Radiotherapy in Metastatic Triple Negative Breast Cancer Patients	- Breast cancer - Metastatic triple negative breast cancer	- Pembrolizumab (200 mg intravenous) (anti-PD-1)	- 30 Gy radiotherapy delivered in 5, 6 Gray (Gy) × 5 fractions	- Phase II - 17 participants - Clinical trial	- Active, not recruiting
**NCT03051672**	A Phase II Study Of Pembrolizumab In Combination With Palliative Radiotherapy For Metastatic Hormone Receptor Positive Breast Cancer	- Metastatic breast cancer	- Pembrolizumab (200 mg intravenous)	- Palliative radiotherapy, 20 Gy in × 5 fractions	- Phase II - 8 participants - Clinical trial	- Terminated
**NCT04756505**	REINA: A Phase I Study of Radiation Enhanced IL 12-Necrosis Attraction in Hormone Receptor Positive, HER2 Negative Metastatic Breast Cancer Patients	- Stage IV breast cancer - Hormone receptor positive breast adenocarcinoma - Metastatic/ metastatic HER2- breast carcinoma - Stage IV breast cancer	- Bintrafusp Alfa (intravenous) - Immunocytokine NHS-IL12 (subcutaneous)	- Radiotherapy	- Phase I - 20 participants - Clinical trial	- Recruiting
**NCT04454528**	Preoperative Use of Radiation Boost to Enhance Effectiveness of Immune Checkpoint Blockade Therapy in Operable Breast Cancer	- Operable breast cancer	- Pembrolizumab (200 mg intravenous)	- Hypofractionated radiotherapy boost of 7 Gy x 1 fraction	- Phase 1b/2 - 27 participants - Clinical trial	- Recruiting
**NCT03366844**	Preoperative Combination of Pembrolizumab and Radiation Therapy in Patients With Operable Breast Cancer	- Breast cancer	- Pembrolizumab	- Radiotherapy boost, 8 Gy x 3 fractions	- Phase I and II - 60 participants - Clinical trial	- Active, not recruiting
**NCT02977468**	Effects of MK-3475 (Pembrolizumab) on the Breast Tumor Microenvironment in Triple Negative Breast Cancer With and Without Intra-operative RT: a Window of Opportunity Study	- Triple negative breast cancer	- Pembrolizumab (MK-3475) (intravenous)	- Intraoperative radiotherapy (IORT) on day of surgery	- Phase I - 15 participants - Clinical trial	- Recruiting
**NCT01862900**	Phase I/II Study of Stereotactic Body Radiation Therapy to Metastatic Lesions in the Liver or Lung in Combination With Monoclonal Antibody to OX40 (MEDI6469) in Patients With Progressive Metastatic Breast Cancer After Systemic Therapy.	- Metastatic breast cancer - Lung metastases -Liver metastases	- Biological: MEDI6469 (anti-OX40) (0.4 mg/kg intravenous)	- Stereotactic body radiotherapy (SBRT) - Three arms: 15 Gy, 20 Gy, or 25 Gy SBRT	- Phase I/II - 14 participants - Clinical trial	- Completed
**NCT04879849**	An Open-label, Phase I, Dose-escalation Study to Evaluate the Safety and Preliminary Antitumor Activity of TAK-676 With Pembrolizumab Following Radiation Therapy in the Treatment of Non-small-cell Lung Cancer, Triple-negative Breast Cancer, or Squamous-cell Carcinoma of the Head and Neck That Has Progressed on Checkpoint Inhibitors	- Triple negative breast neoplasms - Non-small-cell lung carcinoma - Squamous cell carcinoma of head and neck	- Pembrolizumab (200 mg intravenous) - TAK-676 (0.2 mg and above intravenous)	- Image-guided radiotherapy	- Phase I - 65 participants - Clinical trial	- Recruiting
**NCT03807765**	Phase Ib Study of Stereotactic Radiation and Nivolumab in the Management of Metastatic Breast Cancer Brain Metastases	- Metastatic breast cancer brain metastases	- Nivolumab (anti-PD-1) (480 mg intravenous)	- Stereotactic radiosurgery delivered to brain metastases	- Phase I - 14 participants - Clinical trial	- Active, not recruiting
**NCT03818685**	A Multicenter, Randomised, Open-label Phase II Study to Evaluate the Clinical Benefit of a Post-operative Treatment Associating Radiotherapy + Nivolumab + Ipilimumab Versus Radiotherapy + Capecitabine for Triple Negative Breast Cancer Patients With Residual Disease After Neoadjuvant Chemotherapy	- Breast cancer - Triple negative breast neoplasms	- Nivolumab (360 mg intravenous) - Ipilimumab (anti-CTLA4) (1mg/kg intravenous) - Capecitabine (1000mg/m2)	- Radiotherapy delivered per standard practice	- Phase I - 114 participants - Clinical trial	- Active, not recruiting

### The clinical and preclinical promise of combining immunotherapy, radiotherapy, and chemotherapy in breast cancer

**Figure 3:**
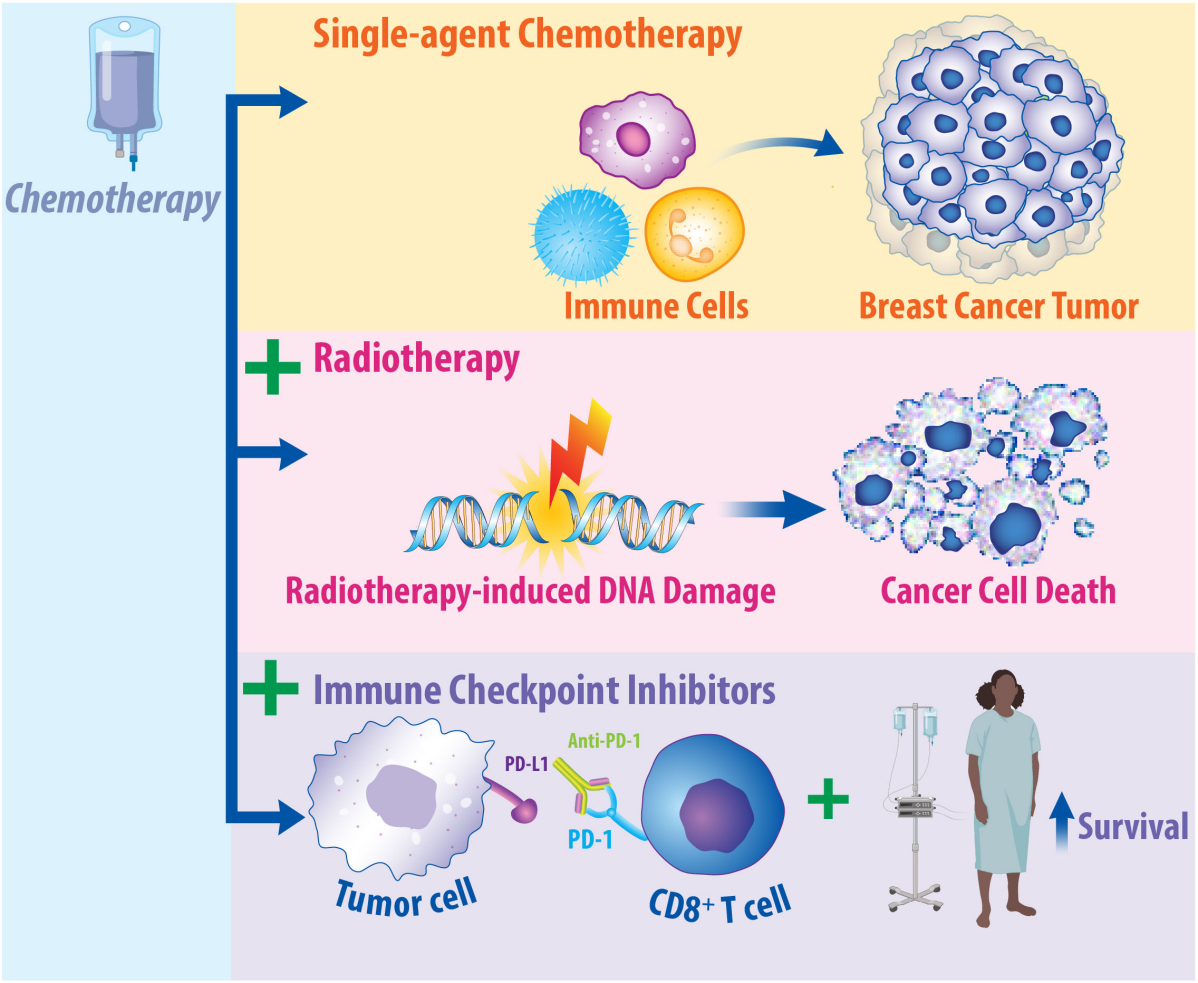
Chemotherapy Has Immunomodulatory Effects on the Tumor Microenvironment and May Promote Synergy in Combination with Radiotherapy and Immune Checkpoint Inhibitors. Chemotherapy is a standard of care therapy for the treatment of breast cancer and has significant implications on the immune response. Studies suggest that single-agent chemotherapy can recruit immune cells to the microenvironment of breast cancer tumors. Additionally, in breast cancer patients, response to chemotherapy is dependent upon the presence of tumor-infiltrating lymphocytes. When chemotherapy is combined with radiotherapy, this can induce radiosensitization in preclinical and clinical models, resulting in enhanced cancer cell death. Clinical promise may exist in combining immune checkpoint inhibitors, radiotherapy, and chemotherapy for the treatment of breast cancer. When chemotherapy is combined with immunotherapy, this enhances its efficacy and increases patient survival. Clinical trials are currently underway to ascertain the effects of combined approaches in breast cancer patients.

Importantly, one potential multimodal therapeutic approach is combining immunotherapy, radiotherapy, and chemotherapy. This approach is summarized in **Figure 3**. The combination of chemotherapy, radiotherapy, and surgery is the standard of care for breast cancer treatment, while numerous studies support the therapeutic potential of combining radiotherapy with chemotherapy for treating breast cancer patients. The evidence supporting the integration of radiotherapy with chemotherapy has been more extensively reviewed elsewhere (140, 195, 196). Importantly, many chemotherapies function by inducing DNA damage, consequently resulting in synergistic effects when combined with radiotherapy in the preclinical and clinical setting (140, 197). Cytotoxic chemotherapeutic agents—such as platinums, taxanes, and antimetabolites—have been found to promote synergistic, radiosensitizing effects in breast cancer (198). Platinum chemotherapies—such as cisplatin and carboplatin—are alkylating agents delivered to breast cancer patients that bind to and crosslink DNA to inhibit proper replication, leading to the formation of double stranded breaks in the DNA (199, 200). Consequently, when platinum therapies are combined with radiotherapy, studies support that this promotes radiosensitization in various subsets of breast cancer, including metastatic IBC (201) and early-stage TNBC (202). Taxanes—such as paclitaxel and docetaxel—inhibit microtubule function, inducing cell cycle arrest at the G2/M Phase, consequently leading to cancer cell death (203). Combining taxane chemotherapy with radiotherapy has been examined in several settings. Combined paclitaxel and radiotherapy led to a 34% complete response in patients with early-stage breast cancer (204). When tested in patients with locoregional recurrence, radiotherapy combined with taxanes or with taxanes combined with cisplatin found increased recurrence-free survival regardless of whether cisplatin was added (205). In the context of locally advanced breast cancer, paclitaxel treatment with concurrent radiotherapy improved disease-free survival and overall survival (206). Antimetabolite chemotherapeutic agents—such as fluoropyridines or gemcitabine—are well-established radiosensitizers that function by mimicking natural metabolites found in the body to become incorporated into DNA or RNA, leading to DNA damage (207, 208). These antimetabolite therapeutics have also been examined in combination with radiotherapy. When treating breast cancer chest wall recurrences with combined gemcitabine and radiotherapy, 100% locoregional control was achieved, although normal tissue toxicity limits this combination clinically (209). Chemotherapy resistant breast cancer treated with capecitabine and radiotherapy was retrospectively analyzed to find that there were no increased toxicities associated with the combination therapy (210). Patients with advanced, non-TNBC treated with capecitabine and radiotherapy led to 73% partial or complete response (211). Collectively, these studies provide the rationale for combining chemotherapy with radiotherapy for the treatment of breast cancer patients.

Chemotherapy, like radiotherapy, has pleotropic effects on the immune system. It is well established that chemotherapy is immunosuppressive, rendering patients undergoing treatment more susceptible to infection (212). However, chemotherapy—particularly in the neoadjuvant setting—has also been found to result in pro-inflammatory, antitumor effects. Neoadjuvant chemotherapy induces immune responses in breast cancer patients, including increasing concentrations of TILs and CD8^+^ T cells (213, 214). Furthermore, the immune response induced by neoadjuvant chemotherapy predicts survival of breast cancer patients and may prime tumors for treatment with immunotherapy (213, 214). The presence of TILs is predictive of response to chemotherapy in breast cancer, further supporting the complex interaction between the immune system and chemotherapy (215). DNA damage immune response signatures have also been confirmed as prognostic biomarkers in TNBC patients treated with adjuvant doxorubicin and cyclophosphamide (216). Additionally, activation of immune responses mediated by the cGAS/STING pathway have been found to predict patient response to neoadjuvant chemotherapy (217). Collectively, these studies support the complex interactions that exist between chemotherapy and the immune system in breast cancer patients. Moreover, these studies also emphasize the importance of further understanding these complex interactions in both preclinical and clinical breast cancer models.

Many clinical trials are currently evaluating the combination of chemotherapy and immunotherapy in breast cancer patients (218). While the focus of this review is trimodal combinations, primarily with radiotherapy, immunotherapy, and additional agents, others have extensively reviewed the effects of combined chemotherapy and immunotherapy (218–220). The I-SPY2 trial (Investigation of Serial Studies to Predict Your Therapeutic Response With Imaging And Molecular Analysis 2) is one such important trial examining ICIs in combination with chemotherapy. This randomized, adaptive clinical trial aims to assess the effects of novel agents combined with standard therapies for stage II or stage III breast cancer patients (NCT01042379) with high-risk MammaPrint scores, a gene signature used to predict breast cancer patient clinical outcomes (221, 222). The primary endpoint for I-SPY 2 is pCR. One arm of I-SPY 2 examined the therapeutic effects of combining pembrolizumab with neoadjuvant chemotherapy in approximately 250 patients. Pembrolizumab more than doubled the pCR rate in the HR^+^, HER2-negative subset (13% to 30%) as well as the TNBC subset (22% to 60%) (223). Jointly, these studies support the clinical promise of combining chemotherapy and immunotherapy.

Clinical trials are currently underway to assess the effectiveness of combining chemotherapy with immunotherapy and/or radiotherapy as summarized in **Table 2**. Trials are currently evaluating the effects of preoperative pembrolizumab combined with neoadjuvant chemotherapy (paclitaxel, carboplatin, cyclophosphamide, doxorubicin, and/or capecitabine) for TNBC or HR^+^, HER2^-^ breast cancer (NCT04443348), in addition to radiotherapy combined with chemotherapy (nab-paclitaxel and paclitaxel) and pembrolizumab in PD-L1^+^ TNBC (NCT05233696). Moreover, a Phase III trial is examining the effects of adjuvant pembrolizumab in combination with radiotherapy on disease-free survival in TNBC patients (NCT02954874). The priming effects of radiotherapy on breast cancer patients prior to neoadjuvant chemotherapy are also being examined to further understand the role of the immune response following radiotherapy (NCT03978663). The TONIC trial is a Phase II, randomized, open-label trial examining whether chemotherapy or radiotherapy prior to immune checkpoint inhibition with nivolumab induces an inflamed tumor microenvironment in metastatic TNBC patients (NCT02499367). In this study, chemotherapy resulted in the most significant patient responses, where cisplatin treated patients had an ORR of 23% and doxorubicin treated patients had an ORR of 35% in addition to an increase in immune cell infiltration. Interestingly, patients pretreated with radiotherapy did not see an increase in immune cell infiltration in the form of CD8^+^ T cells and TILs. However, results from this study suggest that delivering chemotherapy prior to PD-1/PD-L1 inhibition can prime tumors for response to immune checkpoint inhibition (224). These studies highlight the clinical promise of combining chemotherapy, ICIs, and radiotherapy for treating breast cancer patients, and the important research underway to understand the clinical effects of these combined approaches.

**Table 2:** Trials currently assessing combined immune checkpoint inhibition, chemotherapy, and/or radiotherapy.

ClinicalTrials.gov identifier	Study title	Conditions	Therapeutic agent(s)	Radiotherapy	Phase and patients	Status (at time of publication)
**NCT04443348**	P-RAD: A Randomized Study of Preoperative Chemotherapy, Pembrolizumab and No, Low or High Dose RADiation in Node-Positive, HER2-Negative Breast Cancer	- Triple negative breast cancer - Hormone receptor positive breast cancer - Biopsy-proven, positive lymph nodes	- Pembrolizumab - Paclitaxel - Carboplatin - Cyclophosphamide - Doxorubicin - Capecitabine	- Radiotherapy boost	- Phase II - 120 Participants - Clinical trial	- Recruiting
**NCT05233696**	Phase II Study of Radiotherapy in Combination With Chemotherapy and Immunotherapy in Patients With PD-L1-Positive Metastatic Triple-Negative Breast Cancer	- Triple negative breast cancer - Locally advanced breast cancer - Unresectable breast carcinoma - Metastatic breast cancer	- Nab-paclitaxel (100 mg/m2 intravenous) - Paclitaxel (80 mg/m2 intravenous) - Pembrolizumab (200 mg)	- One to four metastatic sites will be treated at the discretion of the radiation oncologist	- Phase II - 29 participants - Clinical trial	- Recruiting
**NCT03978663**	Evaluating the Use of Stereotactic Radiation Therapy Prior to Neoadjuvant Chemotherapy for High-risk Breast Carcinoma (a SIGNAL Series Clinical Trial): Three Fraction Radiation to Induce Immuno-Oncologic Response (TRIO Trial)	- High risk cancer - Locally advanced breast cancer	- Neoadjuvant anthracycline and taxane based chemotherapy	- Neoadjuvant radiotherapy - 8 Gy x 3 fractions, with a fall off dose of 4 Gy x 3 fractions	- N/A - 40 participants - Clinical trial	- Recruiting
**NCT02499367**	Adaptive Phase II Randomized Non-comparative Trial of Nivolumab After Induction Treatment in Triple-negative Breast Cancer (TNBC) Patients: TONIC-trial	- Breast cancer	- Nivolumab(3 mg/kg) - Low dose doxorubicin (15 mg) - Cyclophosphamide (50 mg oral) - Cisplatin (40 mg/m2)	- Radiotherapy; 20 Gy to metastatic lesions	- Phase II - 84 participants - Clinical trial	- Active, not recruiting
**NCT02954874**	A Randomized, Phase III Trial to Evaluate the Efficacy and Safety of Pembrolizumab (MK-3475) as Adjuvant Therapy for Triple Receptor-Negative Breast Cancer With &gt;/= 1 CM Residual Invasive Cancer or Positive Lymph Nodes (ypN1mi, ypN1-3) After Neoadjuvant Chemotherapy	- Invasive breast carcinoma - Stage 0-III breast cancer - Triple negative breast carcinoma	- Pembrolizumab (intravenous)	- Radiotherapy within 12 weeks post treatment or 12 weeks of last breast cancer operation	- Phase III - 1155 participants - Clinical trial	- Active, not recruiting
**NCT02971748**	A Phase II Study of Anti-PD-1 (Pembrolizumab) in Combination With Hormonal Therapy During or After Radiation in Patients With Hormone Receptor (HR)-Positive Localized Inflammatory Breast Cancer (IBC) Who Did Not Achieve a Pathological Complete Response (pCR) to Neoadjuvant Chemotherapy	- Stage III breast cancer - Breast inflammatory carcinoma	- Pembrolizumab (intravenous)	- Radiotherapy	- Phase II - 37 participants - Clinical trial	- Active, not recruiting
**NCT03515798**	A Prospective Multicenter Open-label, Randomized Phase II Study of Pembrolizumab in Combination With Neoadjuvant EC-Paclitaxel Regimen in HER2-negative Inflammatory Breast Cancer.	- HER2-negative, inflammatory breast cancer	- Epirubicine-cyclophosphamide (EC) paclitaxel chemotherapy - Pembrolizumab (MK3475) (intravenous)	- None	- Phase II - 81 participants - Clinical trial	- Recruiting
**NCT05093387**	A Pilot Study of SGT-53 With Carboplatin and Pembrolizumab in Metastatic Triple Negative Inflammatory Breast Cancer	- Metastatic, triple negative inflammatory breast cancer	- Carboplatin (intravenous) - Pembrolizumab (intravenous) - SGT-53 (Transferrin Receptor-Targeted Liposomal p53 cDNA) (intravenous)	- None	- Phase I - 9 participants - Clinical trial	- Not yet recruiting

### The clinical and preclinical promise of combining immunotherapy, radiotherapy, and PARP inhibitors

**Figure 4:**
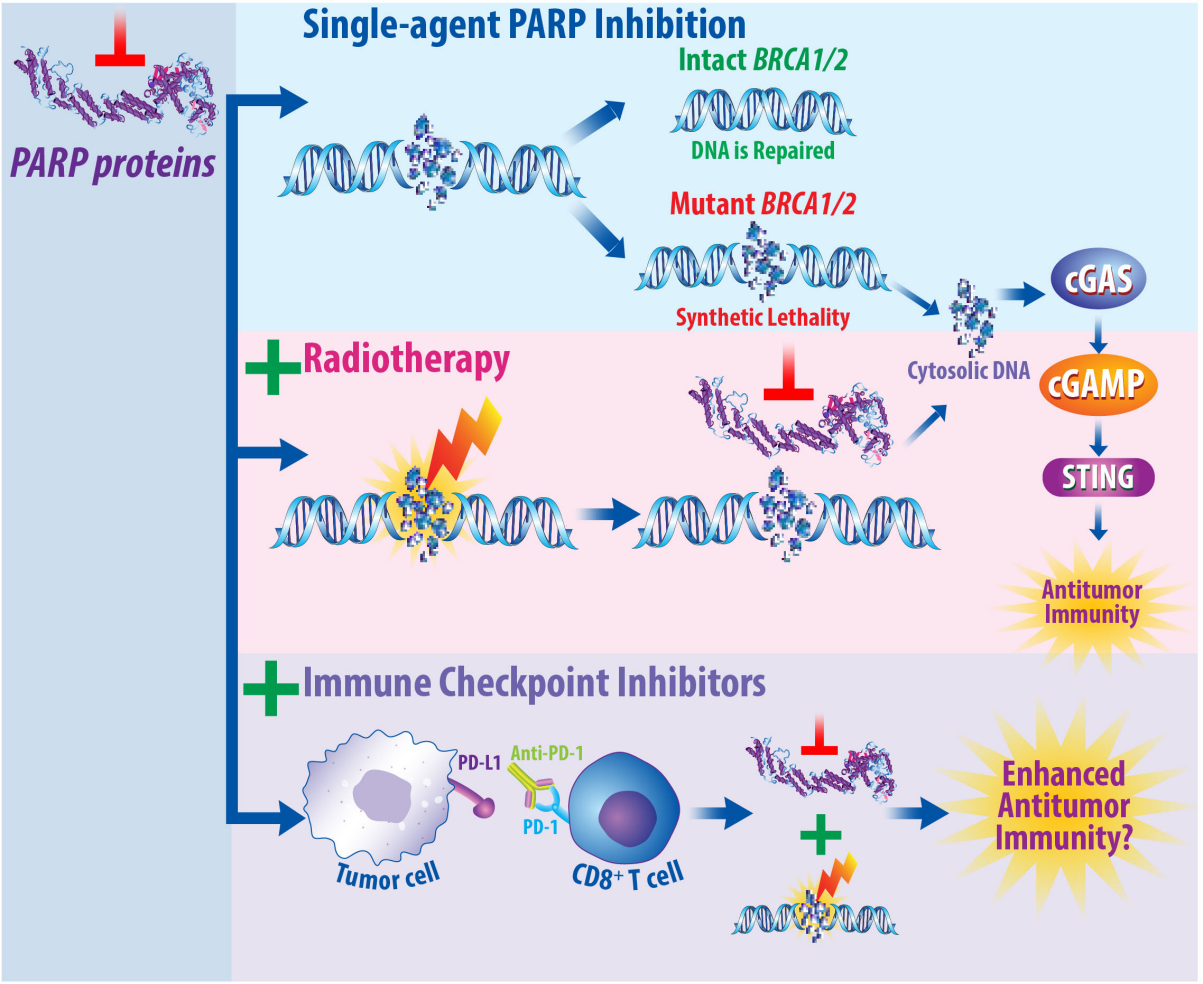
PARP Inhibitors Prevent DNA Damage Repair and May Synergize with Both Radiotherapy and Immune Checkpoint Inhibition. Mechanistically, PARP proteins are recruited to regions of DNA damage to assist in the repair of single-strand breaks. When PARP proteins are inhibited, this prevents proper DNA repair and promotes the accumulation of double-strand breaks. In patients that express the *BRCA1/2* genes, this damage can be repaired; however, in patients with a deleterious *BRCA1/2* mutation, this results in synthetic lethality due to the absence of multiple DNA repair pathways. It is well established that radiotherapy induces DNA damage. When radiotherapy is combined with PARP inhibitors, this prevents DNA damage repair in BRCA mutant cancers. Furthermore, the DNA damage induced by radiotherapy that is then not repaired following PARP inhibition can result in the production of cytosolic DNA molecules. As single agents, immune checkpoint inhibitors illicit immune responses by turning off immune checkpoints, resulting in pro-inflammatory, antitumor effects. Studies are currently underway to determine whether combined PARP inhibition, radiotherapy, and immune checkpoint inhibition will promote enhanced antitumor immunity and be efficacious for the treatment of breast cancer patients.

Another approach for improving the efficacy of immunotherapy exists in combining immunotherapy and radiotherapy with DNA damage inhibitors, as summarized in **Figure 4**. Poly(ADP-ribose) polymerase (PARP) proteins help mediate effective DNA damage responses, and PARP inhibitors hold promise for the treatment of breast cancer by inhibiting this repair process (225). Mechanistically, PARP proteins are recruited to sites of damaged DNA and complete a posttranslational modification termed PARylation (225, 226). PARylation recruits DNA repair proteins to induce repair of single-strand breaks (SSBs) (140, 225, 226). PARP inhibitors prevent the accumulation of DNA damage repair proteins, resulting in increased DNA double-strand breaks (DSBs) (225, 226). Approximately 5% of breast cancer patients carry a deleterious mutation in the Breast Cancer (*BRCA1/2*) genes, which are required for proper DNA damage repair and correlate with increased risk of developing breast cancer (225, 227, 228). In patients with BRCA deleterious mutations, PARP inhibitors cause “synthetic lethality,” wherein loss of multiple DNA repair pathways results in synergistic tumor cell death (229). The PARP inhibitors olaparib and talazoparib are currently FDA-approved for the treatment of HER2-negative, BRCA-mutated breast cancer (225). Combining PARP inhibitors with radiotherapy can promote breast cancer cell death. Mechanistically, radiotherapy induces DNA damage, while PARP inhibitors prevent DNA damage repair (140). PARP1 inhibition was found to radiosensitize breast cancer models to ionizing radiotherapy preclinically (230, 231). Thus, there is a strong preclinical rationale to combine radiotherapy and PARP inhibitors for the treatment of breast cancer clinically.

Clinical trials have begun to evaluate the combination of PARP inhibitors with radiotherapy and/or immunotherapy, which are summarized in **Table 3**. The PARP inhibitor veliparib has been combined with radiotherapy for breast cancer patients with inflammatory disease or locoregionally recurrent disease (NCT01477489) and is currently being examined in breast cancer patients in combination with preoperative radiotherapy (NCT01618357). The PARP inhibitor rucaparib is also currently being investigated in combination with radiotherapy for TNBC patients who do not respond to chemotherapy (NCT03542175). Furthermore, studies are also combining olaparib and radiotherapy (NCT03109080, NCT03598257). For example, the RADIOPARP Phase I trial examined the effects of olaparib combined with 50 Gy radiotherapy for patients with inflammatory, metastatic, or locoregionally advanced TNBC (NCT03109080) (232). While trimodality therapy can cause an increase in acute self-limited adverse events, overall, the combination is well tolerated (233). However, more research is needed to continue monitoring potential toxicities caused by this treatment modality in patients over time (232, 234).

**Table 3:** Clinical trials assessing the effects of PARP inhibitors combined with radiotherapy and/or immune checkpoint inhibitors.

ClinicalTrials.gov identifier	Study title	Conditions	Therapeutic agent(s)	Radiotherapy	Phase and patients	Status (at time of publication)
**NCT01477489**	A Phase I Study of Veliparib Administered Concurrently With Chest Wall and Nodal Radiation Therapy in Patients With Inflammatory or Loco-regionally Recurrent Breast Cancer	- Breast cancer	- Veliparib (50 mg – 200 mg)	- Standard radiotherapy (limited to 60 Gy)	- Phase I - 33 participants - Clinical trial	- Completed
**NCT01618357**	Pre-Operative PARPi and Irradiation (POPI) in Women With an Incomplete Response to Neo-Adjuvant Chemotherapy for Breast Cancer	- Breast cancer	- Lumpectomy/ Mastectomy - Veliparib	- Radiotherapy; 2.35 Gy per fraction for 16 fractions for a total of 37.5 Gy	- Phase I - 41 participants - Clinical trial	- Suspended
**NCT03542175**	A Phase I Study of Rucaparib Administered Concurrently With Postoperative Radiotherapy in Patients With Triple Negative Breast Cancer With an Incomplete Pathologic Response Following Neoadjuvant Chemotherapy	- Breast cancer	- Rucaparib (300 mg, 400 mg, 500 mg, or 600 mg)	- Radiotherapy; 50 Gy in 2 Gy per fraction, plus 10 Gy boost to lumpectomy cavity	- Phase I - 30 participants - Clinical trial	- Recruiting
**NCT03109080**	A Phase I of Olaparib With Radiation Therapy in Patients With Inflammatory, Loco-regionally Advanced or Metastatic TNBC (Triple Negative Breast Cancer) or Patient With Operated TNBC With Residual Disease	- Malignant and triple-negative breast neoplasms	- Olaparib	- Radiotherapy	- Phase I - 24 participants - Clinical trial	- Active, not recruiting
**NCT03598257**	A Phase II Randomized Trial of Olaparib (NSC-747856) Administered Concurrently With Radiotherapy Versus Radiotherapy Alone for Inflammatory Breast Cancer	- Breast inflammatory carcinoma	- Olaparib (oral)	- Radiotherapy	- Phase II - 300 participants - Clinical trial	- Recruiting
**NCT02657889**	Phase 1/2 Clinical Study of Niraparib in Combination With Pembrolizumab (MK-3475) in Patients With Advanced or Metastatic Triple-Negative Breast Cancer and in Patients With Recurrent Ovarian Cancer	- Triple negative breast cancer - Breast cancer - Metastatic breast cancer - Advanced breast cancer - Stage IV breast cancer - Neoplasms - Ovarian cancer - Fallopian tube cancer - Peritoneal cancer	- Niraparib (up to 300 mg/day oral) - Pembrolizumab (200 mg intravenous)	- None	- Phase I/II - 122 participants - Clinical trial	- Completed
**NCT03544125**	A Pilot Study of Olaparib and Durvalumab in Patients With Metastatic Triple Negative Breast Cancer	- Stage IV breast cancer - Estrogen receptor negative - HER2 negative - Progesterone receptor negative - Stage IV breast cancer - Triple-negative breast carcinoma	- Durvalumab (intravenous) - Olaparib (oral)	- None	- Phase I - 3 participants - Clinical trial	- Completed
**NCT03025035**	Open Label, Phase II Pilot Study of Immune Checkpoint Inhibition With Pembrolizumab in Combination With PARP Inhibition With Olaparib in Advanced BRCA-mutated or HDR-defect Breast Cancers	- Breast cancer	- Pembrolizumab (intravenous) - Olaparib (oral)	- None	- Phase II - 20 participants - Clinical trial	- Recruiting
**NCT02849496**	A Phase II Open-Label, Randomized Study of PARP Inhibition (Olaparib) Either Alone or in Combination With Anti-PD-L1 Therapy (Atezolizumab; MPDL3280A) in Homologous DNA Repair (HDR) Deficient, Locally Advanced or Metastatic Non-HER2-Positive Breast Cancer	- Locally advanced - unresectable breast carcinoma - Metastatic breast carcinoma - Stage III breast cancer - Stage IV breast cancer	- Atezolizumab (intravenous) - Olaparib (oral)	- None	- Phase II - 81 participants - Clinical trial	- Suspended
**NCT04683679**	A Phase II Study of Pembrolizumab and Ablative Radiotherapy With or Without Olaparib in Metastatic Triple-Negative Breast Cancers : Initial Test Cohorts of a Platform Trial to Sequentially Investigate Combinations of DNA-Damage Response Inhibitors and Immunotherapy for the Augmentation of Immune Responses	- Triple negative breast cancer	- Pembrolizumab (200 mg intravenous) - Olaparib (600 mg oral)	- 8-9 Gy x 3 fractions or 30 Gy in 6 Gy per fraction for larger tumors	- Phase II - 56 participants - Clinical trial	- Recruiting
**NCT04837209**	A Phase II Study of NirAparib, Dostarlimab and Radiotherapy in Metastatic, PD-L1 Negative or Immunotherapy-Refractory Triple-Negative Breast Cancer (NADiR)	- Breast cancer - Triple negative breast cancer	- Niraparib (oral) - Dostarlimab (anti-PD-1) (intravenous)	- Radiotherapy	- Phase II - 32 participants - Clinical trial	- Recruiting

In addition to contributing to radiation-induced DNA damage, studies also suggest that PARP inhibition regulates antitumor immunity (226). Many studies suggest a connection between *BRCA* mutations, PARP inhibition, and the immune system in breast cancer. In *BRCA*-deficient TNBC models, PARP inhibition with olaparib induces a CD8^+^ T cell response *in vivo* through the activation of the cGAS/STING pathway (235). PARP inhibition also modulates immunosuppressive macrophages in the TME of *BRCA1*-associated TNBC models and treating these models with CSF-1R antibodies combined with PARP inhibitors overcomes PARP inhibitor acquired resistance (236). Moreover, knock down of BRCA2 in human breast cancer cells activates the cGAS/STING pathway (237). Surprisingly, PARP inhibition in some BRCA proficient ovarian and colorectal cancer models can also activate immune responses through the STING pathway (238). Moreover, combining PARP inhibitors with anti-PD-L1 improves tumor control in preclinical breast cancer models (239). These preclinical data suggest that PARP inhibition may promote antitumor immunity.

Furthermore, studies have examined the mechanisms underlying the interactions between resistance to PARP inhibitors and ICIs. PARP inhibitors have been found to upregulate PD-L1 expression, resulting in immunosuppression (240). Glycosylation of PD-L1 is required for its interaction with PD-1 and subsequent suppression of T cell activity (240, 241). However, inhibition of PD-L1 glycosylation via 2-deoxyglucose (2-DG) promotes T-cell mediated cytotoxicity and potent antitumor activity in combination with PARP inhibitors (240). Human and murine TNBC cell lines resistant to PARP inhibitors display an increase in epithelial-mesenchymal transition and upregulation of PD-L1 (242). These effects are abrogated by the application of metformin to block pAkt S473—potentially providing a synergistic approach to increase PARP inhibition and immunotherapy efficacy (242). In short, various studies suggest that PD-L1 upregulation may regulate PARP inhibitor resistance.

Clinical trials are beginning to report the efficacy of PARP inhibition combined with ICIs in breast cancer patients. In the TOPACIO/KEYNOTE-162 trial, the PARP inhibitor niraparib was combined with pembrolizumab for the treatment of advanced or metastatic TNBC (NCT02657889). Preliminary results from this study suggest that combining PARP inhibition with ICIs may be effective in metastatic TNBC regardless of BRCA status (243). Additionally, ongoing studies are examining the combination of olaparib and durvalumab for patients with metastatic TNBC (NCT03544125) (244), as well as examining the combination of pembrolizumab and olaparib in patients with DNA damage response pathway mutations (NCT03025035). Furthermore, a Phase II, open-label, randomized trial was recently underway to assess the effects of olaparib alone and in combination with atezolizumab in HDR deficient locally advanced or metastatic non-HER2^+^ breast cancer, although it was recently suspended (NCT02849496) (245). To conclude, these clinical data suggest that PARP inhibition may enhance patient responses to immunotherapy; however, additional research is merited.

Based upon the promise of combining both PARP inhibition with radiotherapy and PARP inhibition with immunotherapy, trials are also examining trimodal approaches with radiotherapy, ICIs, and PARP inhibition. A Phase II trial is currently recruiting patients to ascertain the efficacy and safety of talazoparib combined with radiotherapy and atezolizumab (anti-PD-L1) for PD-L1^+^ metastatic TNBC patients (NCT04690855). Additionally, a randomized, Phase II study is recruiting breast cancer patients to understand the effects of radiotherapy in combination with pembrolizumab and olaparib to treat patients with triple negative disease (NCT04683679). Moreover, a Phase II trial is currently assessing the effects of combined niraparib, dostarlimab (anti-PD-1), and radiotherapy in metastatic, PD-L1^-^, or immunotherapy-refractory TNBC (NCT04837209). Importantly, more time is necessary to define the tolerability and efficacy of these trimodal approaches in breast cancer patients.

### Safety, tolerability, and cost-effectiveness of combined therapy approaches

Importantly, while combining targeted therapies with radiotherapy and immune checkpoint inhibitors is a promising approach for the treatment of breast cancer patients, more studies are warranted to further examine the safety and tolerance of such combinations. All pharmaceutical agents are associated with potential adverse events and combining therapeutic agents and modalities can heighten the risk of toxicity. Combining therapeutics also has the potential of reducing toxicity if combined therapies are synergistic and require lower doses of these agents in combination compared to when delivered as monotherapies. Clinical and preclinical studies are currently underway to screen for potential adverse effects and unwanted toxicities of combined approaches for the treatment of breast cancer.

Collectively, in breast cancer patients, single agent targeted therapies can result in various toxicities, including cardiovascular (246, 247), endocrine, dermatologic, and pulmonary toxicities (248). While advancements in the delivery of radiotherapy as a monotherapy have allowed for the precise delivery of radiation rays directly to cancerous lesions, radiotherapy can also damage nearby, non-malignant cells, resulting in acute and late-onset toxicities (249). ICIs are associated with idiosyncratic inflammatory adverse events which can occur in potentially any organ system, emphasizing the importance of closely monitoring patients receiving such therapies (250). Anti-CTLA-4 immunotherapies are associated with a higher incidence of immune-related adverse events (irAEs) compared to inhibitors of the PD-1 axis, which may coincide with their different mechanisms of action (249). Anti-PD-1 therapies (i.e., pembrolizumab) may be associated with fewer adverse events than anti-PD-L1 therapies (i.e., atezolizumab) in breast cancer patients (250, 251). Collectively, as more patients receive ICIs as part of their treatment regimens, more screening is warranted to understand why these adverse events take place and how these events can be prevented in patients undergoing treatment.

Combination therapies involving the application of both radiotherapy and ICIs may result in complex effects on the immune system which may promote enhanced therapy efficacy and also therapy toxicity. To date, the combination of radiotherapy and ICIs has been found to be safe and well-tolerated in patients undergoing treatment (249, 252). Combined anti-PD-1 and anti-CTLA-4 ICIs with palliative radiotherapy was found to be associated with few adverse events in patients with non-small cell lung cancer, melanoma, renal cell cancer, and breast cancer (192, 253). Toxicity can also occur in studies combining chemotherapy with ICIs. For instance, in the KEYNOTE-522 trials, while combination of chemotherapy and pembrolizumab improved pathological complete response in patients with early TNBC, this therapy resulted in 78% of patients having grade 3 or higher adverse events, compared to only 73% of patients in the placebo-chemotherapy group (106). Targeted therapy can also cause adverse events. Single-agent PARP inhibition has been found to be less toxic compared to single-agent chemotherapy; however, when PARP inhibitors are used in combination with radiotherapy, toxicity must be closely monitored (248). In a study that combined PARP inhibition (veliparib) with radiotherapy in patients with inflammatory or locoregionally recurrent breast cancer, 1 year post treatment resulted in grade 3 toxicity of 10%. However, 3 years following combined therapy, 46.7% of patients experienced grade 3 toxicity, with 6 out of a total 15 patients having severe fibrosis in the field of treatment (233). Collectively, more studies are needed to screen for such toxicities and determine the proper doses of targeted therapies, ICIs, and radiotherapy that can be efficacious, while inducing minor adverse events and low toxicities in patients with aggressive forms of breast cancer,

While the safety profiles of combined approaches are important to consider when determining the optimal treatment plan, another important aspect to consider is the cost-effectiveness of such therapeutics. Financial toxicity is a growing concern in breast cancer care (254). While ICIs are an emerging and promising therapeutic option for cancer patients, they are costly services for patients, which is a critical factor when patients are deciding what course of therapy to pursue. In a study assessing the cost effectiveness of immunotherapy in non-small cell lung cancer, the median yearly cost of ICIs was $148,431. Importantly, while the costs of ICIs may vary based upon drug rand and mechanism of action, overall, prolonged usage of such therapies beyond two years was not found to be financially feasible for patients (255). Consequently, numerous studies are focused on accessing the cost-effectiveness (CE) of immunotherapies, which is often measured as the incremental cost-effectiveness ratio (ICER), a ratio that represents the cost required for one additional year of life (256). In breast cancer, results from studies assessing the cost-effectiveness of immunotherapies are often mixed and are drug-dependent—supporting the need to further analyze the benefit of prescribing ICIs to cancer patients—especially in combination with other targeted therapies. In solid tumors, ICIs provide significant clinical benefits to patients and certain types of ICIs have been found to be cost-effective in different types of cancer compared to chemotherapy treatment alone (256). In PD-1^+^, metastatic TNBC, the combination of pembrolizumab with chemotherapy was found to be cost-effective (257). Combined chemotherapy and pembrolizumab was also cost-effective in high risk, early-stage TNBC (258). Combining ICIs with radiotherapy is also cost-effective in non-small cell lung cancer; however, this has not been examined as thoroughly in the context of breast cancer and more studies are warranted (259). More work is also necessary to determine how cost-effective trimodal approaches are for breast cancer patients—such as for combined ICIs, radiotherapy, and targeted therapy. Furthermore, this also starts conversations regarding the overall cost of therapeutics and accessibility to affordable healthcare—which may vary based upon where patients are receiving their cancer care and influence their decisions to receive such therapies.

### Future directions

Future clinical trials are focused on assessing whether combination approaches increase immunotherapy efficacy in patients with breast cancer as demonstrated in **Table 4** (260). CDK4/6 inhibitors are mainstay treatments for women with metastatic HR+, HER2^-^ breast cancer and induce radiosensitization in preclinical models of ER^+^ breast cancer and TNBC (261, 262). Furthermore, the CDK4/6 inhibitor abemaciclib enhances the efficacy of anti-PD-L1 ICIs by augmenting antigen presentation and T cell activation in human breast cancer cells (263). These data motivate the assessment of combining CDK4/6 inhibitors with radiotherapy and ICIs in future studies. Currently, the effects of combined stereotactic body radiation (SBRT), ICIs, and hormone therapies are being examined in ER^+^ breast cancer (NCT04563507). In addition to analyzing the effects of already developed pharmacological agents with radiotherapy and ICIs, future studies should investigate the combined effects of novel cancer therapeutic agents. For instance, combining a phosphoinositide 3-kinase δ (PI3Kαδ) inhibitor with radiotherapy and anti-PD-1 was found to increase CD8^+^ T cell accumulation and delay tumor growth in a murine syngeneic TNBC model (264). STING agonists are also currently being examined in preclinical breast cancer models in combination with ubiquitinated protein nanovaccines (265), anti-CD47 monoclonal antibodies (266), and CAR-T cell therapy (267). These studies suggest that combining STING agonists, ICIs, and radiotherapy may have clinical potential.

**Table 4:** Additional studies assessing combinatorial therapies for the treatment of breast cancer.

ClinicalTrials.gov identifier	Study title	Conditions	Therapeutic agent(s)	Radiotherapy	Phase and patients	Status (at time of publication)
**NCT04563507**	CIMER: Combined Immunotherapies in Metastatic ER+ Breast Cancer	- Breast cancer	- Letrozole (2.5 Mg tablet) - Palbociclib (125 mg)	- SBRT at 50 Gy × 5 fractions	- Phase II - 102 participants - Clinical trial	- Recruiting
**NCT04435964**	Gender Difference in sidE eFfects of ImmuNotherapy: a Possible Clue to Optimize cancEr tReatment	- Breast Cancer - Melanoma - Lung cancer - Head and neck cancer - Urogenital neoplasms	- Immune checkpoint inhibitors as a monotherapy or in combination with radiotherapy and/or chemotherapy	- Varies	- 400 participants - Observational trial	- Recruiting
**NCT03383107**	Effect of Radiotherapy Variables on Circulating Effectors of Immune Response and Local Microbiome	- Breast cancer - Prostate cancer	- Radiotherapy	(For breast cancer) - Standard fractionation breast and nodal radiotherapy to 50 Gy in × 25 fractions - Partial breast RT to 30 Gy in × 25 fractions and × 5 fractions	- 66 participants - Observational trial	- Completed
**NCT05037825**	The Gut Microbiome and Immune Checkpoint Inhibitor Therapy in Solid Tumors	- Triple-negative breast cancer - Non-small-cell lung carcinoma - Malignant melanoma - Renal cell carcinoma	- Anti-PD-1, anti-PD-L1, or anti-CTLA-4 in combination with other checkpoint inhibitors or agents including radiotherapy, surgery, and/or chemotherapy	- Varies	- 800 participants - Observational trial	- Recruiting
**NCT01351103**	A Phase I, Open-label, Dose Escalation Study of Oral LGK974 in Patients With Malignancies Dependent on Wnt Ligands	- Triple negative breast cancer - Pancreatic cancer - BRAF mutant colorectal cancer - Melanoma - Head and neck squamous cell cancer - Cervical squamous cell cancer - Esophageal squamous cell cancer - Lung squamous cell cancer	- Drug: LGK974 (PORCN inhibitor) - Biological: PDR001 (anti-PD-1)	- None	- Phase I - 185 participants - Clinical trial	- Recruiting

Additional studies are crucial to determine the most effective radiotherapy dose and fractionation in patients. The optimal dose fractionation to induce effective antitumor immune responses has not yet been determined, with preclinical literature supporting both ablative single fractions (268) as well as moderate hypofractionation (160, 166). For example, ablative stereotactic body radiotherapy delivered at 15 Gy delivered in 3 fractions or 30 Gy radiotherapy delivered in 1 fraction combined with immunotherapy decreased primary tumor size in a 4T1 murine breast cancer model, while ablative radiotherapy delivered at 1 fraction of 30 Gy transforms the tumor suppressive microenvironment of colon tumors into a pro-inflammatory, CD8^+^ T cell enriched environment (268, 269). Hypofractionated radiotherapy delivered at 9.18 Gy in 3 fractions or 6.43 Gy in 5 fractions also induces systemic antitumor effects and promotes synergy in combination with anti-PD-1 in syngeneic breast cancer models (270). Conversely, radiotherapy delivered at doses above 12-18 Gy induces Trex1 in other breast cancer models, which can hinder the pro-immune effects of radiotherapy by degrading cellular DNA upstream of the cGAS/STING pathway (177). Prospective clinical evaluations are needed to define the optimal radiotherapy regimens in patients.

In addition to better understanding the mechanisms involved in radiotherapy, it is also critical to further understand the underlying mechanisms involved in immunotherapy efficacy and patient response to immunotherapy. Importantly, many factors play a role in the efficacy of ICIs, such as age (85), sex (NCT04435964), gut microbiome (NCT03383107, NCT05037825), and oncogenic signaling/mutations (NCT01351103) (271). Immunotherapy efficacy may also depend on sites of metastatic involvement. In both patients and preclinical models, liver metastases are associated with diminished immunotherapy efficacy (272). Moreover, it is essential to continue investigating the effects of the cGAS/STING pathway and its implications in both the radiotherapy response and immune response in human cancers. Numerous studies are currently investigating the preclinical implications of the cGAS/STING pathway in cancer and how other mediators of this pathway can be modulated to promote pro-immune, antitumor effects. In all, the mechanisms underlying combined therapies are complex and more research is justified to further understand these interactions.

Moreover, it is also critical to define treatment tolerance since adverse events may occur following combined treatments. Finally, another crucial future direction is developing predictive and prognostic biomarkers indicative of response to combination therapies. While studies suggest TILs, tumor mutation burden (TMB), and immune gene signatures may be potential biomarkers for response to ICIs in breast cancer, biomarkers indicative of combined therapy efficacy have not yet been identified (273, 274). In short, more research is necessary to discover biomarkers to help identify which patient populations will respond best to these novel therapeutic approaches.

## Discussion

Breast cancer is the leading non-cutaneous cancer diagnosed among females and is a heterogeneous disease that can result in poor clinical outcomes, especially in patients with triple negative disease. Immunotherapy is an emerging therapeutic option for aggressive forms of breast cancer and combining immunotherapy with radiotherapy may hold clinical benefit. Preclinical studies are underway to understand the potential benefit of combining radiotherapy with immune checkpoint inhibitors and to examine the molecular mechanisms that contribute to potential synergy between these therapies. Additional studies are needed to develop therapeutic approaches targeting canonical and noncanonical regulators of innate immunity in conjunction with radiotherapy and immunotherapy. Clinical trials are currently examining the prognostic benefits of combined ICIs and radiotherapy with other available cancer therapeutics in breast cancer patients. Collectively, these studies support the importance of improving combined therapy efficacy with the ultimate goal of improving outcomes in breast cancer.

## Author contributions

KMJ conceptualized this review and drafted the original manuscript. EH, AP, KRJ, CB, LP, MG, and CS contributed to the writing and critical revision. All authors contributed to the article and approved the submitted version.

## Funding

Funding was provided to KMJ by the Pharmacological Sciences Training Program (PSTP) T32 Training Grant (GM007767), the Rackham Merit Fellowship, and the Rackham Graduate School Research Grant. Funding was provided to AP by NIAID Training Grant T32 (AI007413). Funding was provided to MG by the Lung Precision Oncology Program (VA 150CU000182), LUNGevity (2021-07), Veterans Affairs (I01 BX005267), Melanoma Research Alliance (MRA689853), and NCI (CA252010). Support was provided to LP by the Breast Cancer Research Foundation.

## Acknowledgments

We would like to thank Steven Kronenberg at the University of Michigan for his assistance with the graphics in the figures. Graphics for figures were created in part from Biorender.com.

## Conflict of interest

The authors declare that the research was conducted in the absence of any commercial or financial relationships that could be construed as a potential conflict of interest.

## Publisher’s note

All claims expressed in this article are solely those of the authors and do not necessarily represent those of their affiliated organizations, or those of the publisher, the editors and the reviewers. Any product that may be evaluated in this article, or claim that may be made by its manufacturer, is not guaranteed or endorsed by the publisher.

## References

[1] Siegel RL Miller KD Fuchs HE Jemal A. Cancer statistics, 2022. CA: A Cancer J Clin (2022) 72(1):7–33. doi: 10.3322/caac.21708 35020204

[2] Ellington TD Miller JW Henley SJ Wilson RJ Wu M Richardson LC. Trends in breast cancer incidence, by race, ethnicity, and age among women aged ≥20 years — united states, 1999–2018. MMWR Morbidity Mortality Weekly Rep (2022) 71(2):43–7. doi: 10.15585/mmwr.mm7102a2 PMC8757618 35025856

[3] Sachdev JC Sandoval AC Jahanzeb M. Update on precision medicine in breast cancer. In: Von Hoff DD Han H, editors. Precision medicine in cancer therapy, vol. 175 . Gewerbestrasse 11, 6330 Cham, Switzerland: Springer (2019). p. 45–80. doi: 10.1007/978-3-030-16391-4_2 31209841

[4] Prat A Perou CM. Deconstructing the molecular portraits of breast cancer. Mol Oncol (2010) 5(1):5–23. doi: 10.1016/j.molonc.2010.11.003 21147047 PMC5528267

[5] Schettini F Brasó-Maristany F Kuderer NM Prat A. A perspective on the development and lack of interchangeability of the breast cancer intrinsic subtypes. NPJ Breast Cancer (2022) 8(1):1–4. doi: 10.1038/s41523-022-00451-9 35853907 PMC9296605

[6] Almansour NM. Triple-negative breast cancer: A brief review about epidemiology, risk factors, signaling pathways, treatment and role of artificial intelligence. Front Mol Biosci (2022) 9:836417. doi: 10.3389/fmolb.2022.836417 35145999 PMC8824427

[7] Howard FM Olopade OI. Epidemiology of triple-negative breast cancer: A review. Cancer J (2021) 27(1):8–16. doi: 10.1097/PPO.0000000000000500 33475288 PMC12050094

[8] Dietze EC Sistrunk C Miranda-Carboni G O’Regan R Seewaldt VL. Triple-negative breast cancer in African-American women: disparities versus biology. Nat Rev Cancer (2015) 15(4):248–54. doi: 10.1038/nrc3896 PMC5470637 25673085

[9] Osborne CK. Tamoxifen in the treatment of breast cancer. New Engl J Med (1998) 339(22):1609–18. doi: 10.1056/NEJM199811263392207 9828250

[10] Livraghi L Garber JE. PARP inhibitors in the management of breast cancer: current data and future prospects. BMC Med (2015) 13:1–16. doi: 10.1186/s12916-015-0425-1 26268938 PMC4535298

[11] Pernas S Tolaney SM Winder EP Goel S. CDK4/6 inhibition in breast cancer: current practice and future directions. Ther Adv Med Oncol (2018) 10:1–15. doi: 10.1177/1758835918786451 PMC6050811 30038670

[12] Robert C. A decade of immune-checkpoint inhibitors in cancer therapy. Nat Commun (2020) 11(1):1–3. doi: 10.1038/s41467-020-17670-y 32732879 PMC7393098

[13] Mahoney KM Freeman GF McDermott DF. The next immune-checkpoint inhibitors: PD-1/PD-L1 blockade in melanoma. Clin Ther (2015) 37(4):764–82. doi: 10.1016/j.clinthera.2015.02.018 PMC4497957 25823918

[14] Herzberg B Campo MJ Gainor JF. Immune checkpoint inhibitors in non-small cell lung cancer. Oncologist (2017) 22(1):81–8. doi: 10.1634/theoncologist.2016-0189 PMC5313266 27534574

[15] Lopez-Beltran A Cimadamore A Blanca A Massari F Vau N Scarpelli M. Immune checkpoint inhibitors for the treatment of bladder cancer. Cancers (2021) 13(1):1–16. doi: 10.3390/cancers13010131 PMC7795541 33401585

[16] Thomas R Al-Khadairi G Decock J. Immune checkpoint inhibitors in triple negative breast cancer treatment: Promising future prospects. Front Oncol (2021) 10:600573. doi: 10.3389/fonc.2020.600573 33718107 PMC7947906

[17] Haslam A Prasad V. Estimation of the percentage of US patients with cancer who are eligible for and respond to checkpoint inhibitor immunotherapy drugs. JAMA network Open (2019) 2(5):1–9. doi: 10.1001/jamanetworkopen.2019.2535 PMC6503493 31050774

[18] Swoboda A Nanda R. Immune checkpoint blockade for breast cancer. Cancer Treat Res (2018) 173:155–65. doi: 10.1007/978-3-319-70197-4_10 PMC6061922 29349763

[19] Nedeljković M Damjanović A. Mechanisms of chemotherapy resistance in triple-negative breast cancer-how we can rise to the challenge. Cells (2019) 8(9):1–32. doi: 10.3390/cells8090957 PMC6770896 31443516

[20] Wein L Loi S. Mechanisms of resistance of chemotherapy in early-stage triple negative breast cancer (TNBC). Breast (2017) 34:S27–30. doi: 10.1016/j.breast.2017.06.023 28668293

[21] Reits EA Hodge JW Herberts CA Groothuis TA Chakraborty M Wansley EK. Radiation modulates the peptide repertoire, enhances MHC class I expression, and induces successful antitumor immunotherapy. J Exp Med (2006) 203(5):1259–71. doi: 10.1084/jem.20052494 PMC3212727 16636135

[22] Lhuillier C Rudqvist NP Elemento O Formenti SC Demaria S. Radiation therapy and anti-tumor immunity: exposing immunogenic mutations to the immune system. Genome Med (2019) 11(40):1–10. doi: 10.1186/s13073-019-0653-7 31221199 PMC6587285

[23] Amens JN Bahçecioglu G Pinar Z. Immune system effects on breast cancer. Cell Mol Bioengineering (2021) 14(4):279–92. doi: 10.1007/s12195-021-00679-8 PMC8280260 34295441

[24] Pérez-Romero K Rodríguez RM Amedei A Barceló-Coblijn G Lopez DH. Immune landscape in tumor microenvironment: Implications for biomarker development and immunotherapy. Int J Mol Sci (2020) 21(15):1–14. doi: 10.3390/ijms21155521 PMC7432816 32752264

[25] Pai SI Cesano A Marincola FM. The paradox of cancer immune exclusion: Immune oncology next frontier. Cancer Treat Res (2020) 180:173–95. doi: 10.1007/978-3-030-38862-1_6 PMC7423459 32215870

[26] Coussens LM Pollard JW. Leukocytes in mammary development and cancer. Cold Spring Harbor Perspect Biol (2011) 3(3):1–22. doi: 10.1101/cshperspect.a003285 PMC3039933 21123394

[27] Cassetta L Pollard JW. Targeting macrophages: therapeutic approaches in cancer. Nat Rev Drug Discovery (2018) 17(12):887–904. doi: 10.1038/nrd.2018.169 30361552

[28] Lin EY Nguyen AV Russell RG Pollard JW. Colony-stimulating factor 1 promotes progression of mammary tumors to malignancy. J Exp Med (2001) 193(6):727–40. doi: 10.1084/jem.193.6.727 PMC2193412 11257139

[29] Kitamura T Doughty-Shenton D Cassetta L Fragkogianni S Brownlie D Kato Y. Monocytes differentiate to immune suppressive precursors of metastasis-associated macrophages in mouse models of metastatic breast cancer. Front Immunol (2018) 8:2004. doi: 10.3389/fimmu.2017.02004 29387063 PMC5776392

[30] Ramos RN Missolo-Koussou Y Gerber-Ferder Y Bromley CP Bugatti M Núñez NG. Tissue-resident FOLR2 + macrophages associate with CD8 + T cell infiltration in human breast cancer. Cell (2022) 185(7):1189–207. doi: 10.1016/j.cell.2022.02.021 35325594

[31] Ramos RN Rodriguez C Hubert M Ardin M Treilleux I Ries CH. CD163 + tumor-associated macrophage accumulation in breast cancer patients reflects both local differentiation signals and systemic skewing of monocytes. Clin Trans Immunol (2020) 9(2):1–18. doi: 10.1002/cti2.1108 PMC7017151 32082570

[32] Coffelt SB Wellenstein MD de Visser KE. Neutrophils in cancer: neutral no more. Nat Rev Cancer (2016) 16(7):431–46. doi: 10.1038/nrc.2016.52 27282249

[33] Kim IS Gao Y Welte T Wang H Liu J Janghorban M. Immuno-subtyping of breast cancer reveals distinct myeloid cell profiles and immunotherapy resistance mechanisms. Nat Cell Biol (2019) 21(9):1113–26. doi: 10.1038/s41556-019-0373-7 PMC6726554 31451770

[34] Li K Shi H Zhang B Ou X Ma Q Chen Y. Myeloid-derived suppressor cells as immunosuppressive regulators and therapeutic targets in cancer. Signal Transduction Targeted Ther (2021) 6(1):1–25. doi: 10.1038/s41392-021-00670-9 PMC8497485 34620838

[35] Diaz-Montero CM Salem ML Nishimura MI Garrett-Mayer E Cole DJ Montero AJ. Increased circulating myeloid-derived suppressor cells correlate with clinical cancer stage, metastatic tumor burden, and doxorubicin-cyclophosphamide chemotherapy. Cancer Immunology Immunotherapy (2009) 58(1):49–59. doi: 10.1007/s00262-008-0523-4 18446337 PMC3401888

[36] Peng D Tanikawa T Li W Zhao L Vatan L Szeliga W. Myeloid-derived suppressor cells endow stem-like qualities to breast cancer cells through IL6/STAT3 and NO/NOTCH cross-talk signaling. Cancer Res (2016) 76(11):3156–65. doi: 10.1158/0008-5472.CAN-15-2528 PMC4891237 27197152

[37] Basu A Ramamoorthi G Jia Y Faughn J Wiener D Awshah S. Chapter six - immunotherapy in breast cancer: Current status and future directions. In: Wang X-Y Fisher PB, editors. Advances in cancer research. 50 Hampshire Street, 5th Floor, Cambridge, MA 02139, United States: Academic Press (2019). doi: 10.1016/bs.acr.2019.03.006 31202361

[38] Zgura A Galesa L Bratila E Anghel R. Relationship between tumor infiltrating lymphocytes and progression in breast cancer. Maedica (2018) 13(4):317–20. doi: 10.26574/maedica.2018.13.4.317 PMC6362880 30774731

[39] Aftimos P Azim HAJ Sotiriou C. Chapter 26 - molecular biology of breast cancer. In: Coleman WB Tsongalis GJ, editors. Molecular pathology, 2nd ed. 125 London Wall, London EC2Y 5AS, United Kingdom: Academic Press (2018). p. 569–88. doi: 10.1016/B978-0-12-802761-5.00026-2

[40] Adams S Gray RJ Demaria S Goldstain L Perez EA Shulman LN. Prognostic value of tumor-infiltrating lymphocytes in triple-negative breast cancers from two phase III randomized adjuvant breast cancer trials: ECOG 2197 and ECOG 1199. J Clin Oncol (2014) 32(27):2959–66. doi: 10.1200/JCO.2013.55.0491 PMC4162494 25071121

[41] Dieci MV Miglietta F Guarneri V. Immune infiltrates in breast cancer: Recent updates and clinical implications. Cells (2021) 10(2):1–27. doi: 10.3390/cells10020223 PMC7911608 33498711

[42] Tay RET Richardson EK Toh HC. Revisiting the role of CD4 + T cells in cancer immunotherapy–new insights into old paradigms. Cancer Gene Ther (2021) 28(1-2):5–17. doi: 10.1038/s41417-020-0183-x 32457487 PMC7886651

[43] Li C Jiang P Wei S Xu X Wang J. Regulatory T cells in tumor microenvironment: new mechanisms, potential therapeutic strategies and future prospects. Mol Cancer (2020) 19(1):1–23. doi: 10.1186/s12943-020-01234-1 32680511 PMC7367382

[44] Liu S Lachapelle J Leung S Gao D Foulkes WD Nielsen TO. CD8+ lymphocyte infiltration is an independent favorable prognostic indicator in basal-like breast cancer. Breast Cancer Res (2012) 14(2):1–14. doi: 10.1186/bcr3148 PMC3446382 22420471

[45] Egelston CA Avalos C Tu TY Rosario A Wang R Solomon S. Resident memory CD8 + T cells within cancer islands mediate survival in breast cancer patients. JCI Insight (2019) 4(19):1–15. doi: 10.1172/jci.insight.130000 PMC6795408 31465302

[46] Byrne A Savas P Sant S Li R Virassamy B Luen SJ. Tissue-resident memory T cells in breast cancer control and immunotherapy responses. Nat Rev Clin Oncol (2020) 17(6):341–8. doi: 10.1038/s41571-020-0333-y 32112054

[47] Tallón de Lara P Castañón H Vermeer M Núñez N Silina K Sobottka B. CD39 + PD-1 + CD8 + T cells mediate metastatic dormancy in breast cancer. Nat Commun (2021) 12(1):1–14. doi: 10.1038/s41467-021-21045-2 33536445 PMC7859213

[48] Bates GJ Fox SB Han C Leek RD Garcia JF Harris AL. Quantification of regulatory T cells enables the identification of high-risk breast cancer patients and those at risk of late relapse. J Clin Oncol (2006) 24(34):5373–80. doi: 10.1200/JCO.2006.05.9584 17135638

[49] Plitas G Konopacki C Wu K Bos PD Morrow M Putintseva EV. Regulatory T cells exhibit distinct features in human breast cancer. Immunity (2016) 45(5):1122–34. doi: 10.1016/j.immuni.2016.10.032 PMC5134901 27851913

[50] DeNardo DG Coussens LM. Inflammation and breast cancer. balancing immune response: crosstalk between adaptive and innate immune cells during breast cancer progression. Breast Cancer Res (2007) 9(4):1–10. doi: 10.1186/bcr1746 PMC2206719 17705880

[51] Stenmark Tullberg A Puttonen HAJ Sjöström M Holmberg E Chang SL Feng FY. Immune infiltrate in the primary tumor predicts effect of adjuvant radiotherapy in breast cancer; results from the randomized SweBCG91RT trial. Clin Cancer Res (2021) 27(3):749–58. doi: 10.1158/1078-0432.CCR-20-3299 33148672

[52] Chin Y Janseens J Vandepitte J Vandenbrande J Opdebeek L Raus J. Phenotypic analysis of tumor-infiltrating lymphocytes from human breast cancer. Anticancer Res (1992) 12(5):1463–6. 1332579

[53] Ali HR Provenzano E Dawson SJ Blows FM Liu B Shah M. Association between CD8+ T-cell infiltration and breast cancer survival in 12,439 patients. Ann Oncol Off J Eur Soc Med Oncol (2014) 25(8):1536–43. doi: 10.1093/annonc/mdu191 24915873

[54] Marczyk M Qing T O'Meara T Yagahoobi V Pelekanou V Bai Y. Tumor immune microenvironment of self-identified African American and non-African American triple negative breast cancer. NPJ Breast Cancer (2022) 8(1):1–12. doi: 10.1038/s41523-022-00449-3 35869114 PMC9307813

[55] Robertson FM Bondy M Yang W Yamauchi H Wiggins S Kamrudin S. Inflammatory breast cancer: the disease, the biology, the treatment. CA: A Cancer J Clin (2010) 60(6):351–75. doi: 10.3322/caac.20082 20959401

[56] Badr NM McMurray JL Danial I Hayward S Asaad NY Abd El-Wahed MM. Characterization of the immune microenvironment in inflammatory breast cancer using multiplex immunofluorescence. Pathobiology (2022), 1–13. doi: 10.1159/000524549 35705026

[57] Bertucci F Boudin L Finetti P Van Berckelaer C Van Dam P Dirix L. Immune landscape of inflammatory breast cancer suggests vulnerability to immune checkpoint inhibitors. Oncoimmunology (2021) 10(1):e1929724.1–15. doi: 10.1080/2162402X.2021.1929724 PMC8158040 34104544

[58] Hamm CA Moran D Rao K Trusk PB Pry K Sausen M. Genomic and immunological tumor profiling identifies targetable pathways and extensive CD8+/PDL1+ immune infiltration in inflammatory breast cancer tumors. Mol Cancer Ther (2016) 15(7):1746–56. doi: 10.1158/1535-7163.MCT-15-0353 27196778

[59] Van Berckelaer C Van Laere S Colpaert C Bertucci F Kockx M Dirix LY. The immune micro-environment of inflammatory breast cancer is characterized by an influx of CD163+ tumor-associated macrophages. J Clin Oncol (2022)40:2556–6. doi: 10.1200/JCO.2022.40.16_suppl.2556

[60] Valeta-Magara A Gadi A Volta V Walters B Arju R Giashuddin S. Inflammatory breast cancer promotes development of M2 tumor-associated macrophages and cancer mesenchymal cells through a complex chemokine network. Cancer Res (2019) 79(13):3360–71. doi: 10.1158/0008-5472.CAN-17-2158 PMC7331114 31043378

[61] Huang A Cao S Tang L. The tumor microenvironment and inflammatory breast cancer. J Cancer (2017) 8(10):1884–91. doi: 10.7150/jca.17595 PMC5556652 28819386

[62] Ward JP Gubin MM Schreiber RD. The role of neoantigens in naturally occurring and therapeutically induced immune responses to cancer. In: Schreiber RD, editor. Advances in immunology. 50 Hampshire Street, 5th Floor, Cambridge, MA 02139, USA: Academic Press (2016). doi: 10.1016/bs.ai.2016.01.001 PMC6087548 26922999

[63] Jiang T Shi T Zhang H Hu J Song Y Wei J. Tumor neoantigens: from basic research to clinical applications. J Hematol Oncol (2019) 12(1):1–13. doi: 10.1186/s13045-019-0787-5 31492199 PMC6731555

[64] Alexandrov LB Nik-Zainal S Wedge DC Aparicio SAJR Behjati S Biankin AV. Signatures of mutational processes in human cancer. Nature (2013) 500(7463):415–21. doi: 10.1038/nature12477 PMC3776390 23945592

[65] Chen DS Mellman I. Oncology meets immunology: The cancer-immunity cycle. Immunity (2013) 39(1):1–10. doi: 10.1016/j.immuni.2013.07.012 23890059

[66] Boon T Cerottini JC Van den Eynde B van der Bruggen P Van Pel A. Tumor antigens recognized by T lymphocytes. Annu Rev Immunol (1994) 12:337–65. doi: 10.1146/annurev.iy.12.040194.002005 8011285

[67] Benvenuto M Focaccetti C Izzi V Masuelli L Modesti A Bei R. Tumor antigens heterogeneity and immune response-targeting neoantigens in breast cancer. Semin Cancer Biol (2021) 72:65–75. doi: 10.1016/j.semcancer.2019.10.023 31698088

[68] Barroso-Sousa R Jain E Cohen O Kim D Buendia-Buendia J Winer E. Prevalence and mutational determinants of high tumor mutation burden in breast cancer. Ann Oncol (2020) 31(3):387–94. doi: 10.1016/j.annonc.2019.11.010 32067680

[69] Takeshita T Torigoe T Yan L Huang JL Yamashita H Takabe K. The impact of immunofunctional phenotyping on the malfunction of the cancer immunity cycle in breast cancer. Cancers (2020) 13(1):1–17. doi: 10.3390/cancers13010110 33396390 PMC7795596

[70] Hanahan D. Hallmarks of cancer: New dimensions. Cancer Discovery (2022) 12(1):31–46. doi: 10.1158/2159-8290.CD-21-1059 35022204

[71] Bates JP Derakhshandeh R Jones L Webb TJ. Mechanisms of immune evasion in breast cancer. BMC Cancer (2018) 18(1):1–14. doi: 10.1186/s12885-018-4441-3 29751789 PMC5948714

[72] Dhatchinamoorthy K Colbert JD Rock KL. Cancer immune evasion through loss of MHC class I antigen presentation. Front Immunol (2021) 12:636568. doi: 10.3389/fimmu.2021.636568 33767702 PMC7986854

[73] Fang Y Wang L Wan C Sun Y van der Jeught K Zhou Z. MAL2 drives immune evasion in breast cancer by suppressing tumor antigen presentation. J Clin Invest (2021) 131(1):1–17. doi: 10.1172/JCI140837 PMC7773365 32990678

[74] Park S Shi Y Kim BC Jo MH Cruz LO Gou Z. Force-dependent trans-endocytosis by breast cancer cells depletes costimulatory receptor CD80 and attenuates T cell activation. Biosensors Bioelectronics (2020) 165(112389):1–9. doi: 10.1016/j.bios.2020.112389 PMC8011575 32729511

[75] Sun L Wu J Du F Chen X Chen ZJ. Cyclic GMP-AMP synthase is a cytosolic DNA sensor that activates the type I interferon pathway. Science (2013) 339(6121):786–91. doi: 10.1126/science.1232458 PMC3863629 23258413

[76] Barber GN. STING: infection, inflammation and cancer. Nat Rev Immunol (2015) 15(12):760–70. doi: 10.1038/nri3921 PMC5004891 26603901

[77] Guo Q Huang F Goncalves C del Rincón SV Miller WH. “Chapter one - translation of cancer immunotherapy from the bench to the bedside”. In: Fisher PB Wang XY, editors. Advances in cancer research. Academic Press (2019). doi: 10.1016/bs.acr.2019.03.001 31202357

[78] Bakhoum SF Ngo B Laughney AM Cavallo JA Murphy CJ Ly P. Chromosomal instability drives metastasis through a cytosolic DNA response. Nature (2018) 553(7689):467–72. doi: 10.1038/nature25432 PMC5785464 29342134

[79] Kuang X Li J. Chromosome instability and aneuploidy as context-dependent activators or inhibitors of antitumor immunity. Front Immunol (2022) 13, 1–14. doi: 10.3389/fimmu.2022.895961 PMC9393846 36003402

[80] Mackenzie KJ Carroll P Martin CA Murina O Fluteau A Simpson DJ. cGAS surveillance of micronuclei links genome instability to innate immunity. Nature (2017) 548(7668):461–5. doi: 10.1038/nature23449 PMC5870830 28738408

[81] Fuertes MB Woo SR Burnett B Fu YX Gajewski TF. Type I interferon response and innate immune sensing of cancer. Trends Immunol (2013) 34(2):67–73. doi: 10.1016/j.it.2012.10.004 23122052 PMC3565059

[82] Wang Y Luo J Alu A Han X Wei Y Wei X. cGAS-STING pathway in cancer biotherapy. Mol Cancer (2020) 19(1):1–16. doi: 10.1186/s12943-020-01247-w 32887628 PMC7472700

[83] Woo SR Corrales L Gajewski TF. The STING pathway and the T cell-inflamed tumor microenvironment. Trends Immunol (2015) 36(4):250–6. doi: 10.1016/j.it.2015.02.003 PMC4393801 25758021

[84] Parkes EE Humphries MP Gilmore E Sidi FA Bingham V Phyu SM. The clinical and molecular significance associated with STING signaling in breast cancer. NPJ Breast Cancer (2021) 7(1):1–11. doi: 10.1038/s41523-021-00283-z 34172750 PMC8233333

[85] Sceneay J Goreczny GJ Wilson K Morrow S DeCristo MJ Ubellacker JM. Interferon signaling is diminished with age and is associated with immune checkpoint blockade efficacy in triple-negative breast cancer. Cancer Discovery (2019) 9(9):1208–27. doi: 10.1158/2159-8290.CD-18-1454 PMC11167954 31217296

[86] Dane EL Belessiotis-Richards A Backlund C Wang J Hidaka K Milling LE. STING agonist delivery by tumour-penetrating PEG-lipid nanodiscs primes robust anticancer immunity. Nat Materials (2022) 21(6):710–20. doi: 10.1038/s41563-022-01251-z PMC9156412 35606429

[87] Palaia I Tomao F Sassu CM Musacchio L Panici PB. Immunotherapy for ovarian cancer: Recent advances and combination therapeutic approaches. OncoTargets Ther (2020) 13:6109–29. doi: 10.2147/OTT.S205950 PMC7326187 32617007

[88] Retecki K Seweryn M Graczyk-Jarzynka A Bajor A. The immune landscape of breast cancer: Strategies for overcoming immunotherapy resistance. Cancers (2021) 13(23):1–32. doi: 10.3390/cancers13236012 PMC8657247 34885122

[89] Disis ML Cecil DL. Breast cancer vaccines for treatment and prevention. Breast Cancer Res Treat (2022) 191(3):481–9. doi: 10.1007/s10549-021-06459-2 34846625

[90] Guevara ML Persano F Persano S. Advances in lipid nanoparticles for mRNA-based cancer immunotherapy. Front Chem (2020) 8:589959. doi: 10.3389/fchem.2020.589959 33195094 PMC7645050

[91] Miles D Roché H Martin M Perren TJ Cameron DA Glaspy J. Phase III multicenter clinical trial of the sialyl-TN (STn)-keyhole limpet hemocyanin (KLH) vaccine for metastatic breast cancer. Oncologist (2011) 16(8):1092–100. doi: 10.1634/theoncologist.2010-0307 PMC3228158 21572124

[92] Zang X. 2018 Nobel Prize in medicine awarded to cancer immunotherapy: Immune checkpoint blockade - a personal account. Genes Dis (2018) 5(4):302–3. doi: 10.1016/j.gendis.2018.10.003 PMC6303476 30591930

[93] Curiel TJ Wei S Dong H Alvarez X Cheng P Mottram P. Blockade of B7-H1 improves myeloid dendritic cell–mediated antitumor immunity. Nat Med (2003) 9(5):562–7. doi: 10.1038/nm863 12704383

[94] Zou W Wolchok JD Chen L. PD-L1 (B7-H1) and PD-1 pathway blockade for cancer therapy: Mechanisms, response biomarkers, and combinations. Sci Trans Med (2016) 8(328):1–14. doi: 10.1126/scitranslmed.aad7118 PMC4859220 26936508

[95] Lin H Wei S Hurt EM Green MD Zhao L Vatan L. Host expression of PD-L1 determines efficacy of PD-L1 pathway blockade-mediated tumor regression. J Clin Invest (2018) 128(2):805–15. doi: 10.1172/JCI96113 PMC5785251 29337305

[96] Leach DR Krummel MF Allison JP. Enhancement of antitumor immunity by CTLA-4 blockade. Science (1996) 271(5256):1734–6. doi: 10.1126/science.271.5256.1734 8596936

[97] Seidel JA Otsuka A Kabashima K. Anti-PD-1 and anti-CTLA-4 therapies in cancer: Mechanisms of action, efficacy, and limitations. Front Oncol (2018) 8:86(86). doi: 10.3389/fonc.2018.00086 29644214 PMC5883082

[98] Sansom DM. CD28, CTLA-4 and their ligands: who does what and to whom? Immunology (2000) 101(2):169–77. doi: 10.1046/j.1365-2567.2000.00121.x PMC2327073 11012769

[99] Mittendorf EA Philips AV Meric-Bernstam F Qiao N Wu Y Harrington S. PD-L1 expression in triple negative breast cancer. Cancer Immunol Res (2014) 2(4):361–70. doi: 10.1158/2326-6066.CIR-13-0127 PMC4000553 24764583

[100] Davis AA Patel VG. The role of PD-L1 expression as a predictive biomarker: an analysis of all US food and drug administration (FDA) approvals of immune checkpoint inhibitors. J ImmunoTherapy Cancer (2019) 7(1):1–8. doi: 10.1186/s40425-019-0768-9 PMC6815032 31655605

[101] Nanda R Chow LQM Dees EC Berger R Gupta S Geva R. Phase ib KEYNOTE-012 study. J Clin Oncol (2016) 34(21):2460–7. doi: 10.1200/JCO.2015.64.8931 PMC6816000 27138582

[102] Adams S Schmid P Rugo HS Winer EP Loirat D Awada A. Pembrolizumab monotherapy for previously treated metastatic triple-negative breast cancer: cohort a of the phase II KEYNOTE-086 study. Ann Oncol (2019) 30(3):397–404. doi: 10.1093/annonc/mdy517 30475950

[103] Adams S Loi S Toppmeyer D Cescon DW Laurentiis MD Nanda R. Pembrolizumab monotherapy for previously untreated, PD-L1-positive, metastatic triple-negative breast cancer: cohort b of the phase II KEYNOTE-086 stud. Ann Oncol (2019) 30(3):405–11. doi: 10.1093/annonc/mdy518 30475947

[104] Winer EP Lipatov O Im SA Goncalves A Muñoz-Couselo E Lee KS. Pembrolizumab versus investigator-choice chemotherapy for metastatic triple-negative breast cancer (KEYNOTE-119): a randomised, open-label, phase 3 trial. Lancet Oncol (2021) 22(4):499–511. doi: 10.1016/S1470-2045(20)30754-3 33676601

[105] Cortes J Cescon DW Rugo HS Nowecki Z Im SA Yusof MM. Pembrolizumab plus chemotherapy versus placebo plus chemotherapy for previously untreated locally recurrent inoperable or metastatic triple-negative breast cancer (KEYNOTE-355): a randomised, placebo-controlled, double-blind, phase 3 clinical trial. Lancet (2020) 396(10265):1–12. doi: 10.1016/S0140-6736(20)32531-9 33278935

[106] Schmid P Cortes J Pusztai L McArthur H Kümmel S Bergh J. Pembrolizumab for early triple-negative breast cancer. New Engl J Med (2020) 382(9):810–21. doi: 10.1056/NEJMoa1910549 32101663

[107] Cortes J Rugo HS Cescon DW Im SA Yusof MM Gallardo C. Pembrolizumab plus chemotherapy in advanced triple-negative breast cancer. New Engl J Med (2022) 387(3):217–26. doi: 10.1056/NEJMoa2202809 35857659

[108] Berger ER Park T Saridakis A Golshan M Greenup RA Ahuja N. Immunotherapy treatment for triple negative breast cancer. Pharmaceuticals (2021) 14(8):1–13. doi: 10.3390/ph14080763 PMC8401402 34451860

[109] Schmid P Adams S Rugo HS Schneeweiss A Barrios CH Iwata H. Atezolizumab and nab-paclitaxel in advanced triple-negative breast cancer. New Engl J Med (2018) 379(22):2108–21. doi: 10.1056/NEJMoa1809615 30345906

[110] O'Sullivan H Collins D O'Reilly S. Atezolizumab and nab-paclitaxel in advanced triple-negative breast cancer. New Engl J Med (2019) 380(10):985–8. doi: 10.1056/NEJMc1900150 30855755

[111] Rugo HS Delord JP Im SA Ott PA Piha-Paul SA Bedard PL. Safety and antitumor activity of pembrolizumab in patients with estrogen receptor-Positive/Human epidermal growth factor receptor 2-negative advanced breast cancer. Clin Cancer Res (2018) 24(12):2804–11. doi: 10.1158/1078-0432.CCR-17-3452 29559561

[112] Dirix LY Takacs I Jerusalem G Nikolinakos P Arkenau HT Forero-Torres A. Avelumab, an anti-PD-L1 antibody, in patients with locally advanced or metastatic breast cancer: a phase 1b JAVELIN solid tumor study. Breast Cancer Res Treat (2018) 167(3):671–86. doi: 10.1007/s10549-017-4537-5 PMC5807460 29063313

[113] Tolaney SM Barroso-Sousa R Keenan T Li T Trippa L Vaz-Luis I. Effect of eribulin with or without pembrolizumab on progression-free survival for patients with hormone receptor-positive, ERBB2-negative metastatic breast cancer: A randomized clinical trial. JAMA Oncol (2020) 6(10):1598–605. doi: 10.1001/jamaoncol.2020.3524 PMC7489368 32880602

[114] Rugo HS Kabos P Beck JT Chisamore MJ Hossain A Chen Y. A phase ib study of abemaciclib in combination with pembrolizumab for patients with hormone receptor positive (HR+), human epidermal growth factor receptor 2 negative (HER2-) locally advanced or metastatic breast cancer (MBC) (NCT02779751): Interim results. J Clin Oncol (2020) 38, 1051–1. doi: 10.1200/JCO.2020.38.15_suppl.1051

[115] Denkert C von Minckwitz G Darb-Esfahani S Lederer B Heppner BI Weber KE. Tumour-infiltrating lymphocytes and prognosis in different subtypes of breast cancer: a pooled analysis of 3771 patients treated with neoadjuvant therapy. Lancet Oncol (2018) 19(1):40–50. doi: 10.1016/S1470-2045(17)30904-X 29233559

[116] Koch RM Principe DR Cataneo JL Rana A. Progress for immunotherapy in inflammatory breast cancer and emerging barriers to therapeutic efficacy. Cancers (2021) 13(1):1–14. doi: 10.3390/cancers13112543 PMC8196819 34067257

[117] Kharel Z Nemer OP Xi W Upadhayaya B Falkson CI O'Regan RM. Inflammatory breast cancer with excellent response to pembrolizumab-chemotherapy combination: A case report. Breast Dis (2022) 41(1):255–60. doi: 10.3233/BD-210041 35599460

[118] Gao H Kida K Cohen EN Alexander A Lim B Parker C. Abstract P3-09-12: Peripheral T cell clonality and exhaustion as novel biomarkers for anti-PD-1 (pembrolizumab) maintenance therapy in patients with metastatic inflammatory breast cancer (mIBC) and non-IBC triple negative breast cancer (mTNBC). Cancer Res (2020) 80:P3-09. doi: 10.1158/1538-7445.SABCS19-P3-09-12

[119] Wang J Zhou P. New approaches in CAR-T cell immunotherapy for breast cancer. In: Song E Hu H, editors. Translational research in breast cancer, vol. 1026 . Singapore: Springer (2017). p. 371–81. doi: 10.1007/978-981-10-6020-5_17 29282693

[120] Newick K O'Brien S Moon E Albelda SM. CAR T cell therapy for solid tumors. Annu Rev Med (2017) 68:139–52. doi: 10.1146/annurev-med-062315-120245 27860544

[121] June CH Sadelain M. Chimeric antigen receptor therapy. New Engl J Med (2018) 379(1):64–73. doi: 10.1056/NEJMra1706169 29972754 PMC7433347

[122] Lee HJ Kim YA Sim CK Heo SH Song IH Park HS. Expansion of tumor-infiltrating lymphocytes and their potential for application as adoptive cell transfer therapy in human breast cancer. Oncotarget (2017) 8(69):113345–59. doi: 10.18632/oncotarget.23007 PMC5768332 29371915

[123] Morisaki T Kubo M Umebayashi M Yew PY Yoshimura S Park J-H. Neoantigens elicit T cell responses in breast cancer. Sci Rep (2021) 11(1):1–11. doi: 10.1038/s41598-021-91358-1 34193879 PMC8245657

[124] Zacharakis N Huq LM Seitter SJ Kim SP Gartner JJ Sindiri S. Breast cancers are immunogenic: Immunologic analyses and a phase II pilot clinical trial using mutation-reactive autologous lymphocytes. J Clin Oncol (2022) 40(16):1741–54. doi: 10.1200/JCO.21.02170 PMC9148699 35104158

[125] Hauth F Ho AY Ferrone S Duda DG. Radiotherapy to enhance chimeric antigen receptor T-cell therapeutic efficacy in solid tumors: A narrative review. JAMA Oncol (2021) 7(7):1051–9. doi: 10.1001/jamaoncol.2021.0168 PMC8668197 33885725

[126] Katz SC Prince E Cunetta M Guha P Moody A Armenio V. Abstract CT109: HITM-SIR: Phase ib trial of CAR-T hepatic artery infusions and selective internal radiation therapy for liver metastases. Cancer Res (2017) 77:CT109. doi: 10.1158/1538-7445.AM2017-CT109 31155611

[127] Kim KW Jeong JU Lee KH Uong TNT Rhee JH Ahn SJ. Combined NK cell therapy and radiation therapy exhibit long-term therapeutic and antimetastatic effects in a human triple negative breast cancer model. Int J Radiat Oncology Biology Phys (2020) 108(1):115–25. doi: 10.1016/j.ijrobp.2019.09.041 31605787

[128] Li P Yang L Li T Bin S Sun B Huang Y. The third generation anti-HER2 chimeric antigen receptor mouse T cells alone or together with anti-PD1 antibody inhibits the growth of mouse breast tumor cells expressing HER2 *in vitro* and in immune competent mice. Front Oncol (2020) 10:1143. doi: 10.3389/fonc.2020.01143 32766150 PMC7381237

[129] Nigro CL Macagno M Sangiolo D Bertolaccini L Aglietta M Merlano MC. NK-mediated antibody-dependent cell-mediated cytotoxicity in solid tumors: biological evidence and clinical perspectives. Ann Trans Med (2019) 7(5):1–12. doi: 10.21037/atm.2019.01.42 PMC6462666 31019955

[130] Camejo N Castillo C Alonso R Rivero E Mezquita C Rosich A. Efficacy of trastuzumab for HER-2 positive breast cancer in a real-life setting: A decade of experience under national treatment coverage regulations. J Clin Oncol (2018) 36(15):e18789. doi: 10.1200/JCO.2018.36.15_suppl.e18789 PMC7051800 32045546

[131] Modi S Jacot W Yamashita T Sohn J Vidal M Tokunaga E. Trastuzumab deruxtecan in previously treated HER2-low advanced breast cancer. New Engl J Med (2022) 387(1):9–20. doi: 10.1056/NEJMoa2203690 35665782 PMC10561652

[132] Cadena A Cushman TR Anderson CL Barsoumian HB Welsh JW. Cortez MA radiation and anti-cancer vaccines: A winning combination. Vaccines (2018) 6(1):1–8. doi: 10.3390/vaccines6010009 PMC5874650 29385680

[133] Formenti SC Demaria S. Combining radiotherapy and cancer immunotherapy: a paradigm shift. J Natl Cancer Institute (2013) 105(4):256–65. doi: 10.1093/jnci/djs629 PMC3576324 23291374

[134] Mignot F Ajgal Z Xu H Geraud A Chen JY Mégnin-Chanet F. Concurrent administration of anti-HER2 therapy and radiotherapy: Systematic review. Radiotherapy Oncol (2017) 124(2):190–9. doi: 10.1016/j.radonc.2017.07.006 28751231

[135] Minn I Rowe SP Pomper MG. Enhancing CAR T-cell therapy through cellular imaging and radiotherapy. Lancet Oncol (2019) 20(8):e443–51. doi: 10.1016/S1470-2045(19)30461-9 31364596

[136] Grubbé EH. Priority in the therapeutic use of X-rays. Radiology (1933) 21(2):156–62. doi: 10.1148/21.2.156

[137] Castaneda SA Strasser J. Updates in the treatment of breast cancer with radiotherapy. Surg Oncol Clinics North America (2017) 26(3):371–82. doi: 10.1016/j.soc.2017.01.013 28576177

[138] Murshed H. “Chapter 3 - radiation biology”. In: Murshed H, editor. Fundamentals of radiation oncology. 125 London Wall, London EC2Y 5AS, United Kingdom: Academic Press (2019). doi: 10.1016/B978-0-12-814128-1.00003-9

[139] Formenti SC Demaria S. Systemic effects of local radiotherapy. Lancet Oncol (2009) 10(7):718–26. doi: 10.1016/S1470-2045(09)70082-8 PMC2782943 19573801

[140] Pesch AM Pierce LJ Speers CW. Modulating the radiation response for improved outcomes in breast cancer. JCO Precis Oncol (2021) 5:245–64. doi: 10.1200/PO.20.00297 PMC8232831 34250414

[141] E. B. C. T. C. G. (EBCTCG) Darby S McGale P Correa C Taylor C Arriagada R. Effect of radiotherapy after breast-conserving surgery on 10-year recurrence and 15-year breast cancer death: meta-analysis of individual patient data for 10 801 women in 17 randomised trials. Lancet (2011) 378(9804):1707–16. doi: 10.1016/S0140-6736(11)61629-2 PMC3254252 22019144

[142] Poortmans P. Evidence based radiation oncology: breast cancer. Radiotherapy Oncol J Eur Soc Ther Radiol Oncol (2007) 84(1):84–101. doi: 10.1016/j.radonc.2007.06.002 17599597

[143] E. B. C. T. C. G. (EBCTCG). Effects of radiotherapy and of differences in the extent of surgery for early breast cancer on local recurrence and 15-year survival: an overview of the randomised trials. Lancet (2005) 366(9503):2087–106. doi: 10.1016/S0140-6736(05)67887-7 16360786

[144] Quast U. Whole body radiotherapy: A TBI-guideline. J Med Phys (2006) 31(1):5–12. doi: 10.4103/0971-6203.25664 21206634 PMC3003894

[145] Mole RH. Whole body irradiation–radiobiology or medicine? Br J Radiol (1953) 26(305):234–41. doi: 10.1259/0007-1285-26-305-234 13042090

[146] Seiwert TY Kiess AP. Time to debunk an urban myth? the "Abscopal effect" with radiation and anti-PD-1. J Clin Oncol (2020) 39(1):1–4. doi: 10.1200/JCO.20.02046 32986527

[147] Kaminski JM Shinohara E Summers JB Niermann KJ Morimoto A Brousal J. The controversial abscopal effect. Cancer Treat Rev (2005) 31(3):159–72. doi: 10.1016/j.ctrv.2005.03.004 15923088

[148] Hu ZI McArthur HL Ho AY. The abscopal effect of radiation therapy: What is it and how can we use it in breast cancer? Curr Breast Cancer Rep (2017) 9(1):45–51. doi: 10.1007/s12609-017-0234-y 28344743 PMC5346418

[149] Golden EB Chhabra A Chachoua A Adams S Donach M Fenton-Kerimian M. Local radiotherapy and granulocyte-macrophage colony-stimulating factor to generate abscopal responses in patients with metastatic solid tumours: a proof-of-principle trial. Lancet Oncol (2015) 16(7):795–803. doi: 10.1016/S1470-2045(15)00054-6 26095785

[150] Demaria S Ng B Devitt ML Babb JS Kawashima N Liebes L. Ionizing radiation inhibition of distant untreated tumors (abscopal effect) is immune mediated. Int J Radiat Oncology Biology Phys (2004) 58(3):862–70. doi: 10.1016/j.ijrobp.2003.09.012"10.1016/j.ijrobp.2003.09.012 14967443

[151] Ngwa W Irabor OC Schoenfeld JD Hesser J Demaria S Formenti SC. Using immunotherapy to boost the abscopal effect. Nat Rev Cancer (2018) 18:313–22. doi: 10.1038/nrc.2018.6 PMC5912991 29449659

[152] McBride S Sherman E Tsai CJ Baxi S Aghalar J Eng J. Randomized phase II trial of nivolumab with stereotactic body radiotherapy versus nivolumab alone in metastatic head and neck squamous cell carcinoma. J Clin Oncol (2021) 1):30–7. doi: 10.1200/JCO.20.00290 PMC8462641 32822275

[153] Stone HB Peters LJ Milas L. Effect of host immune capability on radiocurability and subsequent transplantability of a murine fibrosarcoma. J Natl Cancer Institute (1979) 63(5):1229–35. doi: 10.1093/jnci/63.5.1229 291749

[154] Mansfield AS Park SS Dong H. Synergy of cancer immunotherapy and radiotherapy. Aging (2015) 7(3):144–5. doi: 10.18632/aging.100730 PMC4394723 25868470

[155] Gong J Le TQ Massarelli E Hendifar AE Tuli R. Radiation therapy and PD-1/PD-L1 blockade: the clinical development of an evolving anticancer combination. J Immunotherapy Cancer (2018) 6(1):1–17. doi: 10.1186/s40425-018-0361-7 PMC5987486 29866197

[156] Stump CT Roehle K Manjarrez Orduno N Dougan SK. Radiation combines with immune checkpoint blockade to enhance T cell priming in a murine model of poorly immunogenic pancreatic cancer. Open Biol (2021) 11(11):1–10. doi: 10.1098/rsob.210245 PMC8595997 34784792

[157] Oweida A Lennon S Calame D Korpela S Bhatia S Sharma J. Ionizing radiation sensitizes tumors to PD-L1 immune checkpoint blockade in orthotopic murine head and neck squamous cell carcinoma. Oncoimmunology (2017) 6(10):1–10. doi: 10.1080/2162402X.2017.1356153 PMC5665079 29123967

[158] Pilones KA Joseph A Vatner R Formenti S Demaria S. Radiation therapy sensitizes a poorly immunogenic breast cancer to PD-1 blockade. Int J Radiat Oncology Biology Phys (2014) 90(1):S58. doi: 10.1016/j.ijrobp.2014.05.203

[159] Demaria S Kawashima N Yang AM Devitt ML Babb JS Allison JP. Immune-mediated inhibition of metastases after treatment with local radiation and CTLA-4 blockade in a mouse model of breast cancer. Clin Cancer Res (2005) 11(2):728–34. doi: 10.1158/1078-0432.728.11.2 15701862

[160] Dewan MZ Galloway AE Kawashima N Dewyngaert JK Babb JS Formenti SC. Fractionated but not single-dose radiotherapy induces an immune-mediated abscopal effect when combined with anti-CTLA-4 antibody. Clin Cancer Res (2009) 15(17):5379–88. doi: 10.1158/1078-0432.CCR-09-0265 PMC2746048 19706802

[161] Pilones KA Kawashima N Yang AM Babb JS Formenti SC Demaria S. Invariant natural killer T cells regulate breast cancer response to radiation and CTLA-4 blockade. Clin Cancer Res (2009) 15(2):597–606. doi: 10.1158/1078-0432.CCR-08-1277 19147765 PMC2730222

[162] Matsumura S Wang B Kawashima N Braunstein S Badura M Cameron TO. Radiation-induced CXCL16 release by breast cancer cells attracts effector T cells. J Immunol (2008) 181(5):3099–107. doi: 10.4049/jimmunol.181.5.3099 PMC2587101 18713980

[163] Verbrugge I Gasparini A Haynes NM Hagekyriakou J Galli M Stewart TJ. The curative outcome of radioimmunotherapy in a mouse breast cancer model relies on mTOR signaling. Radiat Res (2014) 182(2):219–29. doi: 10.1667/RR13511.1 24960417

[164] Aguilera T Rafat M Kariolis M von Eyben R Graves E Giaccia A. Tumor immunologic heterogeneity influences response to radiation and combination immunotherapy. J ImmunoTherapy Cancer (2015) 3:345. doi: 10.1186/2051-1426-3-S2-P345

[165] Dovedi SJ Cheadle EJ Popple AL Poon E Morrow M Stewart R. Fractionated radiation therapy stimulates antitumor immunity mediated by both resident and infiltrating polyclonal T-cell populations when combined with PD-1 blockade. Clin Cancer Res (2017) 23(18):5514–26. doi: 10.1158/1078-0432.CCR-16-1673 28533222

[166] Ye JC Formenti SC. Integration of radiation and immunotherapy in breast cancer - treatment implications. Breast (2017) 38:66–74. doi: 10.1016/j.breast.2017.12.005 29253718

[167] Lussier DM Alspach E Ward JP Miceli AP Runci D White JM. Radiation-induced neoantigens broaden the immunotherapeutic window of cancers with low mutational loads. Proc Natl Acad Sci United States America (2021) 118(24):1–9. doi: 10.1073/pnas.2102611118 PMC8214694 34099555

[168] Golden EB Frances D Pellicciotta I Demaria S Barcellos-Hoff MH Formenti SC. Radiation fosters dose-dependent and chemotherapy-induced immunogenic cell death. Oncoimmunology (2014) 3:e285181–12. doi: 10.4161/onci.28518 PMC4106151 25071979

[169] Lee Y Auh SL Wang Y Burnette B Wang Y Meng Y. Therapeutic effects of ablative radiation on local tumor require CD8+ T cells: changing strategies for cancer treatment. Blood (2009) 114(3):589–95. doi: 10.1182/blood-2009-02-206870 PMC2713472 19349616

[170] Lang X Green MD Wang W Yu J Choi JE Jiang L. Radiotherapy and immunotherapy promote tumoral lipid oxidation and ferroptosis via synergistic repression of SLC7A11. Cancer Discovery (2019) 9(12):1673–85. doi: 10.1158/2159-8290.CD-19-0338 PMC6891128 31554642

[171] Zhang Q Green MD Lang X Lazarus J Parsels JD Wei S. Inhibition of ATM increases interferon signaling and sensitizes pancreatic cancer to immune checkpoint blockade therapy. Cancer Res (2019) 79(15):3940–51. doi: 10.1158/0008-5472.CAN-19-0761 PMC6684166 31101760

[172] Wang W McMillan MT Zhao X Wang Z Jiang L Karnak D. DNA-PK inhibition and radiation promote anti-tumoral immunity through RNA polymerase III in pancreatic cancer. Mol Cancer Res (2022) 20(7):1137–50. doi: 10.1158/1541-7786.MCR-21-0725 PMC9262824 35348737

[173] Deng L Liang H Xu M Yang X Burnette B Arina A. STING-dependent cytosolic DNA sensing promotes radiation-induced type I interferon-dependent antitumor immunity in immunogenic tumors. Immunity (2014) 45(5):843–52. doi: 10.1016/j.immuni.2014.10.019 PMC5155593 25517616

[174] Storozynsky Q Hitt MM. The impact of radiation-induced DNA damage on cGAS-STING-Mediated immune responses to cancer. Int J Mol Sci (2020) 21(8877):1–22. doi: 10.3390/ijms21228877 PMC7700321 33238631

[175] Hayman TJ Baro M MacNeil T Phoomak C Aung TN Cui W. STING enhances cell death through regulation of reactive oxygen species and DNA damage. Nat Commun (2021) 12(1):1–14. doi: 10.1038/s41467-021-22572-8 33875663 PMC8055995

[176] Carozza JA Böhnert V Nguyen KC Skariah G Shaw KE Brown JA. Extracellular cGAMP is a cancer cell-produced immunotransmitter involved in radiation-induced anti-cancer immunity. Nat Cancer (2020) 1(2):184–96. doi: 10.1038/s43018-020-0028-4 PMC7990037 33768207

[177] Vanpouille-Box C Alard A Aryankalayil MJ Sarfraz Y Diamond JM Schneider RJ. DNA Exonuclease Trex1 regulates radiotherapy-induced tumour immunogenicity. Nat Commun (2017) 8(1):1–15. doi: 10.1038/ncomms15618 28598415 PMC5472757

[178] Deng L Liang H Burnette B Beckett M Darga T Weichselbaum RR. Irradiation and anti-PD-L1 treatment synergistically promote antitumor immunity in mice. J Clin Invest (2014) 124(2):687–95. doi: 10.1172/JCI67313 PMC3904601 24382348

[179] Twyman-Saint Victor C Rech AJ Maity A Rengan R Pauken KE E. Stelekati E. Radiation and dual checkpoint blockade activate non-redundant immune mechanisms in cancer. Nature (2015) 520(7547):373–7. doi: 10.1038/nature14292 PMC4401634 25754329

[180] Sharabi AB Nirschl CJ Kochel CM Nirschl TR Francica BJ Velarde E. Stereotactic radiation therapy augments antigen-specific PD-1–mediated antitumor immune responses via cross-presentation of tumor antigen. Cancer Immunol Res (2015) 3(4):345–55. doi: 10.1158/2326-6066.CIR-14-0196 PMC4390444 25527358

[181] Song HN Jin H Kim JH Ha IB Kang KM Choi HS. Abscopal effect of radiotherapy enhanced with immune checkpoint inhibitors of triple negative breast cancer in 4T1 mammary carcinoma model. Int J Mol Sci (2021) 22(19):1–9. doi: 10.3390/ijms221910476 PMC8509046 34638817

[182] Gallucci S Maffei ME. DNA Sensing across the tree of life. Trends Immunol (2017) 10:719–32. doi: 10.1016/j.it.2017.07.012 28886908

[183] Stetson DB Medzhitov R. Recognition of cytosolic DNA activates an IRF3-dependent innate immune response. Immunity (2006) 24(1):93–103. doi: 10.1016/j.immuni.2005.12.003 16413926

[184] Ren Z Ding T Zuo Z Xu Z Deng J Wei Z. Regulation of MAVS expression and signaling function in the antiviral innate immune response. Frontiers in Immunology (2020) 11:1–12. doi: 10.3389/fimmu.2020.01030 32536927 PMC7267026

[185] Elion DL Jacobson ME Hicks DJ Rahman B Sanchez V Gonzales-Ericsson PI. Therapeutically active RIG-1 agonist induces immunogenic tumor cell killing in breast cancers. Cancer Res (2018) 78(21):6183–95. doi: 10.1158/0008-5472.CAN-18-0730 30224377

[186] Burleigh K Maltbaek JH Cambier S Green R Gale M James RC. Human DNA-PK activates a STING-independent DNA sensing pathway. Sci Immunol (2020) 5(43):1–13. doi: 10.1126/sciimmunol.aba4219 PMC7081723 31980485

[187] Bhatelia K Singh K Singh R. TLRs: linking inflammation and breast cancer. Cell Signaling (2014) 26(11):2350–7. doi: 10.1016/j.cellsig.2014.07.035 25093807

[188] Kang TH Mao CP Kim YS Kim TW Yang A Lam B. TLR9 acts as a sensor for tumor-released DNA to modulate anti-tumor immunity after chemotherapy. J ImmunoTherapy Cancer (2019) 7(1):1–8. doi: 10.1186/s40425-019-0738-2 PMC6794732 31619293

[189] Dongye Z Li J Wu Y. Toll-like receptor 9 agonists and combination therapies: strategies to modulate the tumour immune microenvironment for systemic anti-tumour immunity. Br J Cancer (2022) 1–11. doi: 10.1038/s41416-022-01876-6 PMC9333350 35902641

[190] Younes AI Barsoumian HB Sezen D Verma V Patel R Wasley M. Addition of TLR9 agonist immunotherapy to radiation improves systemic antitumor activity. Trans Oncol (2021) 14(2):1–7. doi: 10.1016/j.tranon.2020.100983 PMC7750418 33340886

[191] Wang S Campos J Gallotta M Gong M Crain C Naik E. Intratumoral injection of a CpG oligonucleotide reverts resistance to PD-1 blockade by expanding multifunctional CD8+ T cells. Proc Natl Acad Sci (2016) 113(46):E7240–9. doi: 10.1073/pnas.1608555113 PMC5135381 27799536

[192] Ho AY Barker CA Arnold BB Powell SN Hu ZI Gucalp A. A phase 2 clinical trial assessing the efficacy and safety of pembrolizumab and radiotherapy in patients with metastatic triple-negative breast cancer. Cancer (2020) 126(4):850–60. doi: 10.1002/cncr.32599 31747077

[193] Barroso-Sousa R Krop IE Trippa L Tan-Wasielewski Z Li T Osmani W. A phase II study of pembrolizumab in combination with palliative radiotherapy for hormone receptor-positive metastatic breast cancer. Clin Breast Cancer (2020) 20(3):238–45. doi: 10.1016/j.clbc.2020.01.01 32113750

[194] Verbus EA Rossi AJ Clark AS Taunk NK Nayak A Hernandez JM. Preoperative use of a radiation boost to enhance effectiveness of immune checkpoint blockade therapy in operable breast cancer. Ann Surg Oncol (2022) 29(3):1530–2. doi: 10.1245/s10434-021-10987-y 34783947

[195] Garg AK Buchholz TA. Influence of neoadjuvant chemotherapy on radiotherapy for breast cancer. Ann Surg Oncol (2015) 22(5):1434–40. doi: 10.1245/s10434-015-4402-x 25727554

[196] Dubey AK Recht A Come S Shulman L Harris J. Why and how to combine chemotherapy and radiation therapy in breast cancer patients. Recent Results Cancer Res (1998) 152:247–54. doi: 10.1007/978-3-642-45769-2_23 9928562

[197] Fard AE Tavakoli MB Salehi H Emami H. Synergetic effects of docetaxel and ionizing radiation reduced cell viability on MCF-7 breast cancer cell. Appl Cancer Res (2017) 37(29):1–12. doi: 10.1186/s41241-017-0035-7

[198] Brackstone M Palma D Tuck AB Scott L Potvin K Vandenberg T. Concurrent neoadjuvant chemotherapy and radiation therapy in locally advanced breast cancer. Int J Radiat Oncology Biology Phys (2017) 99(4):769–76. doi: 10.1016/j.ijrobp.2017.06.005 28870785

[199] Decatris MP Sundar S O'Byrne KJ. Platinum-based chemotherapy in metastatic breast cancer: current status. Cancer Treat Rev (2004) 30(1):53–81. doi: 10.1016/S0305-7372(03)00139-7 14766126

[200] Zhou J Kang Y Chen L Wang H Liu J Zeng S. The drug-resistance mechanisms of five platinum-based antitumor agents. Front Pharmacol (2020) 11:343. doi: 10.3389/fphar.2020.00343 32265714 PMC7100275

[201] Genet D Lejeune C Bonnier P Aubard Y Venat-Bouvet L Adjadj DJ. Concomitant intensive chemoradiotherapy induction in non-metastatic inflammatory breast cancer: long-term follow-up. Br J Cancer (2007) 97(7):883–7. doi: 10.1038/sj.bjc.6603987 PMC2360400 17876327

[202] Bellon JR Chen YH Rees R Taghian AJ Wong JS Punglia RS. A phase 1 dose-escalation trial of radiation therapy and concurrent cisplatin for stage II and III triple-negative breast cancer. Int J Radiat Oncology Biology Phys (2021) 111(1):45–52. doi: 10.1016/j.ijrobp.2021.03.002 33713742

[203] Gradishar WJ. Taxanes for the treatment of metastatic breast cancer. Breast Cancer Basic Clin Res (2012) 6:159–71. doi: 10.4137/BCBCR.S8205 PMC3486789 23133315

[204] Chakravarthy AB Kelley MC McLaren B Truica CI Billheimer D Mayer IA. Neoadjuvant concurrent paclitaxel and radiation in stage II/III breast cancer. Clin Cancer Res (2006) 12(5):1570–6. doi: 10.1158/1078-0432.CCR-05-2304 16533783

[205] Semrau S Gerber B Reimer T Klautke G Fietkau R. Concurrent radiotherapy and taxane chemotherapy in patients with locoregional recurrence of breast cancer. A retrospective analysis. Strahlentherapie und Onkologie (2006) 182(10):596–603. doi: 10.1007/s00066-006-1549-1 17013573

[206] Adams S Chakravarthy AB Donach M Spicer D Lymberis S Singh B. Preoperative concurrent paclitaxel-radiation in locally advanced breast cancer: pathologic response correlates with five-year overall survival. Breast Cancer Res Treat (2010) 124(3):723–32. doi: 10.1007/s10549-010-1181-8 PMC3655407 20878462

[207] Peters GJ. Novel developments in the use of antimetabolites. Nucleosides Nucleotides Nucleic Acids (2014) 33(4-6):358–74. doi: 10.1080/15257770.2014.894197 24940694

[208] Shewach DS Lawrence TS. Antimetabolite radiosensitizers. J Clin Oncol (2007) 25(26):4043–50. doi: 10.1200/JCO.2007.11.5287 17827452

[209] Suh WW Schott AF Hayman JA Schipper MJ Shewach DS Pierce LJ. A phase I dose escalation trial of gemcitabine with radiotherapy for breast cancer in the treatment of unresectable chest wall recurrences. Breast J (2004) 10(3):204–10. doi: 10.1111/j.1075-122X.2004.21305.x 15125746

[210] Sherry AD Mayer IA Ayala-Peacock DN Abramson VG Rexer BN Chakravarthy AB. Combining adjuvant radiotherapy with capecitabine in chemotherapy-resistant breast cancer: Feasibility, safety, and toxicity. Clin Breast Cancer (2020) 20(4):344–52. doi: 10.1016/j.clbc.2020.02.010 32234364

[211] Woodward WA Fang P Arriaga L Gao H Cohen EN Reuben JM. A phase 2 study of capecitabine and concomitant radiation in women with advanced breast cancer. Int J Radiat Oncology Biology Phys (2017) 99(4):777–83. doi: 10.1016/j.ijrobp.2017.04.030 PMC6072264 28843370

[212] Rasmussen L Arvin A. Chemotherapy-induced immunosuppression. Environ Health Perspect (1982) 43:21–5. doi: 10.1289/ehp.824321 PMC1568884 7037385

[213] Park YH Lal S Lee JE Choi YL Wen J Ram S. Chemotherapy induces dynamic immune responses in breast cancers that impact treatment outcome. Nat Commun (2020) 11(1):1–14. doi: 10.1038/s41467-020-19933-0 33268821 PMC7710739

[214] Ladoire S Mignot G Dabakuyo S Arnould L Apetoh L Rébé C. *In situ* immune response after neoadjuvant chemotherapy for breast cancer predicts survival. J Pathol (2011) 224(3):389–400. doi: 10.1002/path.2866 21437909

[215] Denkert C Loibl S Noske A Roller M Müller BM Komor M. Tumor-associated lymphocytes as an independent predictor of response to neoadjuvant chemotherapy in breast cancer. J Clin Oncol (2010) 28(1):105–13. doi: 10.1200/JCO.2009.23.7370 19917869

[216] Sharma P Barlow WE Godwin AK Parkes EE Knight LA Walker SM. Validation of the DNA damage immune response signature in patients with triple-negative breast cancer from the SWOG 9313c trial. J Clin Oncol (2019) 37(36):3484–92. doi: 10.1200/JCO.19.00693 PMC7194448 31657982

[217] Parkes EE Savage KI Lioe T Boyd C Halliday S Walker SM. Activation of a cGAS-STING-mediated immune response predicts response to neoadjuvant chemotherapy in early breast cancer. Br J Cancer (2021) 126(2):247–58. doi: 10.1038/s41416-021-01599-0 PMC8770594 34728791

[218] Esteva FJ Hubbard-Lucey VM Tang J Pusztai L. Immunotherapy and targeted therapy combinations in metastatic breast cancer. Lancet Oncol (2019) 20(3):e175–86. doi: 10.1016/S1470-2045(19)30026-9 30842061

[219] Cyprian FS Akhtar S Gatalica Z Vranic S. Targeted immunotherapy with a checkpoint inhibitor in combination with chemotherapy: A new clinical paradigm in the treatment of triple-negative breast cancer. Bosnian J Basic Med Sci (2019) 19(3):227–33. doi: 10.17305/bjbms.2019.4204 PMC6716092 30915922

[220] Xin Y Shen G Zheng Y Guan Y Huo X Li J. Immune checkpoint inhibitors plus neoadjuvant chemotherapy in early triple-negative breast cancer: a systematic review and meta-analysis. BMC Cancer (2021) 21(1):1–10. doi: 10.1186/s12885-021-08997-w 34814874 PMC8609839

[221] Wang H Yee D. I-SPY 2: a neoadjuvant adaptive clinical trial designed to improve outcomes in high-risk breast cancer. Curr Breast Cancer Rep (2019) 11(4):303–10. doi: 10.1007/s12609-019-00334-2 PMC7731787 33312344

[222] Cardoso F van't Veer LJ Bogaerts J Slaets L Viale G Delaloge S. 70-gene signature as an aid to treatment decisions in early-stage breast cancer. New Engl J Med (2016) 375:717–29. doi: 10.1056/NEJMoa1602253 27557300

[223] Nanda R Liu MC Yau C Shatsky R Pusztai L Wallace A. Effect of pembrolizumab plus neoadjuvant chemotherapy on pathologic complete response in women with early-stage breast cancer: An analysis of the ongoing phase 2 adaptively randomized I-SPY2 trial. JAMA Oncol (2020) 6(5):676–84. doi: 10.1001/jamaoncol.2019.6650 PMC7058271 32053137

[224] Voorwerk L Slagter M Horlings HM Sikorska K van de Vijver KK de Maaker M. Immune induction strategies in metastatic triple-negative breast cancer to enhance the sensitivity to PD-1 blockade: the TONIC trial. Nat Med (2019) 25(6):920–8. doi: 10.1038/s41591-019-0432-4 31086347

[225] Cortesi L HS Jackisch C. An overview of PARP inhibitors for the treatment of breast cancer. Targeted Oncol (2021) 16(3):255–82. doi: 10.1007/s11523-021-00796-4 PMC8105250 33710534

[226] Césaire M Thariat J Candéias SM Stefan D Saintigny Y Chevalier F. Combining PARP inhibition, radiation, and immunotherapy: A possible strategy to improve the treatment of cancer? Int J Mol Sci (2018) 19(12):1–18. doi: 10.3390/ijms19123793 PMC6321381 30487462

[227] Peto J Collins N Barfoot R Seal S Warren W Rahman N. Prevalence of BRCA1 and BRCA2 gene mutations in patients with early-onset breast cancer. J Natl Cancer Institute (1999) 91(11):943–9. doi: 10.1093/jnci/91.11.943 10359546

[228] Kuchenbaecker KB Hopper JL Barnes DR Phillips KA Mooij TM Roos-Blom MJ. Risks of breast, ovarian, and contralateral breast cancer for BRCA1 and BRCA2 mutation carriers. JAMA (2017) 317(23):2402–16. doi: 10.1001/jama.2017.7112 28632866

[229] Helleday T. The underlying mechanism for the PARP and BRCA synthetic lethality: clearing up the misunderstandings. Mol Oncol (2011) 5(4):387–93. doi: 10.1016/j.molonc.2011.07.001 PMC5528309 21821475

[230] Michmerhuizen AR Pesch AM Moubadder L Chandler BC Wilder-Romans K Cameron M. PARP1 inhibition radiosensitizes models of inflammatory breast cancer to ionizing radiation. Mol Cancer Ther (2019) 18(11):2063–73. doi: 10.1158/1535-7163.MCT-19-0520 PMC6825563 31413177

[231] Feng FY Speers C Liu M Jackson WC Moon D Rinkinen J. Targeted radiosensitization with PARP1 inhibition: optimization of therapy and identification of biomarkers of response in breast cancer. Breast Cancer Res Treat (2014) 147(1):81–94. doi: 10.1007/s10549-014-3085-5 25104443

[232] Loap P Loirat D Berger F Ricci F Vincent-Salomon A Ezzili C. Combination of olaparib and radiation therapy for triple negative breast cancer: Preliminary results of the RADIOPARP phase 1 trial. Int J Radiat Oncology Biology Phys (2021) 109(2):436–40. doi: 10.1016/j.ijrobp.2020.09.032 32971187

[233] Jagsi R Griffith KA Bellon JR Woodward WA Horton JK Ho A. Concurrent veliparib with chest wall and nodal radiotherapy in patients with inflammatory or locoregionally recurrent breast cancer: The TBCRC 024 phase I multicenter study. J Clin Oncol (2018) 36(13):1317–22. doi: 10.1200/JCO.2017.77.2665 PMC6804907 29558281

[234] Loap P Loirat D Berger F Cao K Ricci F Jochem A. Combination of olaparib with radiotherapy for triple-negative breast cancers: One-year toxicity report of the RADIOPARP phase I trial. Int J Cancer (2021) 140(10):1828–32. doi: 10.1002/ijc.33737 34270809

[235] Pantelidou C Sonzogni O De Oliveria Taveira M Mehta AK Kothari A Wang D. PARP inhibitor efficacy depends on CD8 + T-cell recruitment via intratumoral STING pathway activation in BRCA-deficient models of triple-negative breast cancer. Cancer Discovery (2019) 9(6):722–37. doi: 10.1158/2159-8290.CD-18-1218 PMC6548644 31015319

[236] Mehta AK Cheney EM Hartl CA Pantelidou C Oliwa M Castrillon JA. Targeting immunosuppressive macrophages overcomes PARP inhibitor resistance in BRCA1-associated triple-negative breast cancer. Nat Cancer (2021) 2(1):66–82. doi: 10.1038/s43018-020-00148-7 33738458 PMC7963404

[237] Reisländer T Lombardi EP Groelly FJ Miar A Porru M Di Vito S. BRCA2 abrogation triggers innate immune responses potentiated by treatment with PARP inhibitors. Nat Commun (2019) 10(1):1–13. doi: 10.1038/s41467-019-11048-5 31316060 PMC6637138

[238] Shen J Zhao W Ju Z Wang L Peng Y Labrie M. PARPi triggers the STING-dependent immune response and enhances the therapeutic efficacy of immune checkpoint blockade independent of BRCAness. Cancer Res (2019) 79(2):311–9. doi: 10.1158/0008-5472.CAN-18-1003 PMC6588002 30482774

[239] Jiao S Xia W Yamaguchi H Wei Y Chen MK Hsu JM. PARP inhibitor upregulates PD-L1 expression and enhances cancer-associated immunosuppression. Clin Cancer Res (2017) 23(14):3711–20. doi: 10.1158/1078-0432.CCR-16-3215 PMC5511572 28167507

[240] Shao B Li CW Lim SO Sun L Lai YJ Hou J. Deglycosylation of PD-L1 by 2-deoxyglucose reverses PARP inhibitor-induced immunosuppression in triple-negative breast cancer. Am J Cancer Res (2018) 8(9):1837–46. PMC6176188 30323975

[241] Li CW Lim SO Xia W Lee HH Chan LC Kuo CW. Glycosylation and stabilization of programmed death ligand-1 suppresses T-cell activity. Nat Commun (2016) 7:1–11. doi: 10.1038/ncomms12632 PMC5013604 27572267

[242] Han Y Li CW Hsu JM Hsu JL Chan LC Tan X. Metformin reverses PARP inhibitors-induced epithelial-mesenchymal transition and PD-L1 upregulation in triple-negative breast cancer. Am J Cancer Res (2019) 9(4):800–15. PMC6511636 31106005

[243] Vinayak S Tolaney SM Schwartzberg LS Mita MM McCann GAL Tan AR. TOPACIO/Keynote-162: Niraparib + pembrolizumab in patients (pts) with metastatic triple-negative breast cancer (TNBC), a phase 2 trial. J Clin Oncol (2018) 36(16):1011–1. doi: 10.1200/JCO.2018.36.15_suppl.1011

[244] Li A Labrie M Vuky J Lim JY Johnson B Sivagnanam S. Feasibility of real-time serial comprehensive tumor analytics: Pilot study of olaparib and durvalumab in metastatic triple negative breast cancer (mTNBC). J Clin Oncol (2020) 38:e13092. doi: 10.1200/JCO.2020.38.15_suppl.e13092

[245] LoRusso P Pilat MJP Santa-Maria CA Connolly RM Roesch EE Afghahi A. A phase II open-label, randomized study of PARP inhibition (olaparib) either alone or in combination with anti-PD-L1 therapy (atezolizumab) in homologous DNA repair (HDR) deficient, locally advanced or metastatic non-HER2-positive breast cancer. J Clin Oncol (2020) 38(15):TPS1102. doi: 10.1200/JCO.2020.38.15_suppl.TPS1102

[246] Chen DH Tyebally S Malloupas M Roylance R Spurrell E Raja F. Cardiovascular disease amongst women treated for breast cancer: Traditional cytotoxic chemotherapy, targeted therapy, and radiation therapy. Curr Cardiol Rep (2021) 23(3):1–9. doi: 10.1007/s11886-021-01446-x 33501515

[247] Khouri MG Douglas PS Mackey JR Martin M Scott JM Scherrer-Crosbie M. Cancer therapy-induced cardiac toxicity in early breast cancer: Addressing the unresolved issues. Circulation (2012) 126(23):2749–63. doi: 10.1161/CIRCULATIONAHA.112.100560 PMC3667651 23212997

[248] Anders CK LeBoeus NR Bashoura L Faiz SA Shariff AI Thomas A. What’s the price? toxicities of targeted therapies in breast cancer care. Am Soc Clin Oncol Educ Book (2020) 40:55–70. doi: 10.1200/EDBK_279465 32421449

[249] Hwang WL Pike LRG Royce TJ Mahal BA Loeffler JS. Safety of combining radiotherapy with immune-checkpoint inhibition. Nat Rev Clin Oncol (2018) 15(8):477–94. doi: 10.1038/s41571-018-0046-7 29872177

[250] Criscitiello C Corti C Pravettoni G Curigliano G. Managing side effects of immune checkpoint inhibitors in breast cancer. Crit Rev Oncology/Hematology (2021) 162:1–12. doi: 10.1016/j.critrevonc.2021.103354 34029683

[251] Zou Y Zou X Zheng S Tang H Zhang L Liu P. Efficacy and predictive factors of immune checkpoint inhibitors in metastatic breast cancer: a systematic review and meta-analysis. Ther Adv Med Oncol (2020) 12:1–17. doi: 10.1177/1758835920940928 PMC7436841 32874208

[252] Anscher MS Arora S Weinstock C Amatya A Bandaru P Tang C. Association of radiation therapy with risk of adverse events in patients receiving immunotherapy: A pooled analysis of trials in the US food and drug administration database. JAMA Oncol (2022) 8(2):232–40. doi: 10.1001/jamaoncol.2021.6439 PMC8739815 34989781

[253] Bang A Wilhite TJ Pike LRG Cagney DN Aizer AA Taylor A. Multicenter evaluation of the tolerability of combined treatment with PD-1 and CTLA-4 immune checkpoint inhibitors and palliative radiation therapy. Int J Radiat Oncology Biology Phys (2017) 98(2):344–51. doi: 10.1016/j.ijrobp.2017.02.003 28463153

[254] Gharzai LA Ryan KA Szczygiel L Goold S Smith G Hawley S. Financial toxicity during breast cancer treatment: A qualitative analysis to inform strategies for mitigation. JCO Oncol Pract (2021) 17(10):e1413–23. doi: 10.1200/OP.21.00182 34251880

[255] Guirgis H. Costs of extended use of the immune checkpoint inhibitors in first-line non-small cell lung cancer. J Clin Pathways (2021) 7(10):32–6. doi: 10.25270/jcp.2021.12.4

[256] Verma V Sprave T Haque W Simone CB Chsng JY Welsh JM. A systematic review of the cost and cost-effectiveness studies of immune checkpoint inhibitors. J ImmunoTherapy Cancer (2018) 6(1):1–15. doi: 10.1186/s40425-018-0442-7 PMC6251215 30470252

[257] Huang M Fasching P Haiderali A Pan W Gray E Zhou ZY. Cost-effectiveness of pembrolizumab plus chemotherapy as first-line treatment in PD-L1-positive metastatic triple-negative breast cancer. Immunotherapy (2022) 14(13):1027–41. doi: 10.2217/imt-2022-0082 35796042

[258] Fasching PA Huang M Haiderali A Xue W Pan W Zhou ZY. 99P cost effectiveness of pembrolizumab in combination with chemotherapy as neoadjuvant therapy and continued as a single agent as adjuvant therapy for high-risk early-stage TNBC in the united states. Ann Oncol (2022) 33:S170–1. doi: 10.1016/j.annonc.2022.03.115

[259] Giuliani J Fiorica F. Cost-effectiveness of immune checkpoint inhibitors and radiotherapy in advanced non-small cell lung cancer. J Oncol Pharm Pract (2021) 8):2004–6. doi: 10.1177/10781552211038925 34558362

[260] Nguyen AT Shiao SL McArthur HL. Advances in combining radiation and immunotherapy in breast cancer. Clin Breast Cancer (2021) 21(2):143–52. doi: 10.1016/j.clbc.2021.03.007 PMC8562891 33810972

[261] Pesch AM Hirsh NH Michmerhuizen AR Jungles KM Wilder-Romans K Chandler BC. RB expression confers sensitivity to CDK4/6 inhibitor-mediated radiosensitization across breast cancer subtypes. JCI Insight (2022) 7(3):1–18. doi: 10.1172/jci.insight.154402 PMC8855810 34932500

[262] Pesch AM Hirsh NH Chandler BC Michmerhuizen AR Ritter CL Androsiglio MP. Short-term CDK4/6 inhibition radiosensitizes estrogen receptor–positive breast cancers. Clin Cancer Res Off J Am Assoc Cancer Res (2020) 26(24):6568–80. doi: 10.1158/1078-0432.CCR-20-2269 PMC7744368 32967938

[263] Schaer DA Beckmann RP Dempsey JA Huber L Forest A Amaladas N. The CDK4/6 inhibitor abemaciclib induces a T cell inflamed tumor microenvironment and enhances the efficacy of PD-L1 checkpoint blockade. Cell Rep (2018) 22(11):2978–94. doi: 10.1016/j.celrep.2018.02.053 29539425

[264] Chang WI Han MG Kang MH Park JM Kim EE Bae J. PI3Kαδ inhibitor combined with radiation enhances the antitumor immune effect of anti-PD1 in a syngeneic murine triple-negative breast cancer model. Int J Radiat Oncology Biology Phys (2021) 110(3):845–58. doi: 10.1016/j.ijrobp.2021.01.025 33642128

[265] Huang F Pan N Wei Y Zhao J Aldarouish M Wang X. Effects of combinatorial ubiquitinated protein-based nanovaccine and STING agonist in mice with drug-resistant and metastatic breast cancer. Front Immunol (2021) 12:707298. doi: 10.3389/fimmu.2021.707298 34589084 PMC8475273

[266] Kosaka A Ishibashi K Nagato T Kitamura H Fujiwara Y Yasuda S. CD47 blockade enhances the efficacy of intratumoral STING-targeting therapy by activating phagocytes. J Exp Med (2021) 218(11):1–16. doi: 10.1084/jem.20200792 PMC8480673 34559187

[267] Xu N Palmer DC Robeson AC Shou P Bommiasamy H Laurie SJ. STING agonist promotes CAR T cell trafficking and persistence in breast cancer. J Exp Med (2021) 218(2):1–16. doi: 10.1084/jem.20200844 PMC7780733 33382402

[268] Filatenkov A Baker J Mueller AMS Kenkel J Ahn GO Dutt S. Ablative tumor radiation can change the tumor immune cell microenvironment to induce durable complete remissions. Clin Cancer Res (2015) 21(16):3727–39. doi: 10.1158/1078-0432.CCR-14-2824 PMC4537844 25869387

[269] Wang S Kuczma M Pi W Kong V Campbell J Jin JY. Combined stereotactic body radiation therapy and immunotherapy on 4T1 triple-negative breast cancer murine model. Int J Radiat Oncology Biology Phys (2016) 96(2):1–9. doi: 10.1016/j.ijrobp.2016.06.2088

[270] Zhang X Niedermann G. Abscopal effects with hypofractionated schedules extending into the effector phase of the tumor-specific T-cell response. Int J Radiat Oncology Biology Phys (2018) 101(1):63–73. doi: 10.1016/j.ijrobp.2018.01.094 29534901

[271] Sharma P Hu-Lieskovan S Wargo JA Ribas A. Primary, adaptive, and acquired resistance to cancer immunotherapy. Cell (2017) 168(4):707–23. doi: 10.1016/j.cell.2017.01.017 PMC5391692 28187290

[272] Yu J Green MD Li S Sun Y Journey SN Choi JE. Liver metastasis restrains immunotherapy efficacy via macrophage-mediated T cell elimination. Nat Med (2021) 27(1):152–64. doi: 10.1038/s41591-020-1131-x PMC8095049 33398162

[273] Isaacs J Anders C McArthur H Force J. Biomarkers of immune checkpoint blockade response in triple-negative breast cancer. Curr Treat Options Oncol (2021) 22(5):1–15. doi: 10.1007/s11864-021-00833-4 33743085

[274] Sivapiragasam A Ashok Kumar P Sokol ES Albacker LA Killian JK Ramkissoon SH. Predictive biomarkers for immune checkpoint inhibitors in metastatic breast cancer. Cancer Med (2021) 10(1):53–61. doi: 10.1002/cam4.3550 33314633 PMC7826457

